# Cathepsin S: a double-edged protease in barrier damage, neuroimmune inflammation, and tissue repair, with implications for dermatology

**DOI:** 10.3389/fimmu.2026.1937685

**Published:** 2026-10-05

**Authors:** Haien Wu, Fang Su, Chang Li, Chunfang Guo, Zhen Wang

**Affiliations:** 1 Department of Dermatology, The Seventh People’s Hospital of Shenyang, Shenyang, China; 2 Department of Gastroenterology, The People’s Hospital of Liaoning Province, Shenyang, China

**Keywords:** barrier dysfunction, cathepsin S, extracellular matrix, neuroimmune inflammation, skin disease, wound healing

## Abstract

Cathepsin S (CTSS) is a lysosomal cysteine protease traditionally recognized for its role in invariant-chain degradation and major histocompatibility complex class II antigen presentation. However, unlike many lysosomal cathepsins, CTSS remains active at neutral or mildly acidic pH, enabling proteolytic functions at the cell surface and within extracellular inflammatory microenvironments. This property makes CTSS particularly relevant to skin biology, where barrier disruption, immune activation, neuroimmune signaling, extracellular matrix remodeling, angiogenesis, fibrosis, tumor progression, and tissue repair often coexist. This review summarizes the context-dependent and double-edged role of CTSS in inflammation and repair, with emphasis on its dermatological relevance. CTSS can promote pathogenic inflammation through multiple mechanisms, including regulation of antigen presentation, activation of cytokine pathways such as IL-36γ, degradation of antimicrobial and barrier-associated proteins, activation of protease-activated receptor 2, cleavage of fractalkine/CX3CL1, and modulation of neuroimmune circuits involved in itch and pain. These mechanisms implicate CTSS in inflammatory and immune-mediated dermatoses such as psoriasis, atopic dermatitis, vitiligo, rosacea, seborrheic dermatitis, and cutaneous lupus-related autoimmunity. CTSS may also contribute to extracellular matrix turnover, fibroblast activation, vascular remodeling, skin aging, keloid formation, systemic sclerosis-associated fibrosis, and cutaneous melanoma progression. Conversely, CTSS is not uniformly pathogenic. In selected contexts, it may support macrophage, M2 polarization, matrix remodeling, wound repair, and inflammatory resolution. This duality suggests that the biological impact of CTSS depends on its timing, location, cellular source, substrate specificity, and disease stage. Recent pharmacological findings, including rebound CTSS activity and increased CTSS protein mass after repeated administration of a selective CTSS inhibitor, further indicate that simple systemic inhibition may be insufficient or potentially counterproductive. Therefore, CTSS should be viewed as a context-dependent protease switch rather than a universally harmful enzyme. A better understanding of its compartment-specific and stage-specific functions may help guide more selective therapeutic strategies, including local delivery, substrate-selective modulation, combination approaches, and disease-stage-adapted intervention. This framework may refine the development of CTSS-targeted therapies for skin diseases and skin-related immune disorders.

## 1 Introduction

Proteolysis is an irreversible post-translational mechanism that can reshape protein structure, stability, localization and interactions, thereby functioning as a decisive molecular switch in inflammation, immunity, tissue remodeling and host defense (1, 2). Among proteases, cathepsin S (CTSS) is of particular interest to dermatology because its activity is not restricted to acidic lysosomes. Unlike many lysosomal cathepsins, CTSS retains enzymatic activity at neutral or mildly acidic pH, allowing it to function at the cell surface and within the extracellular inflammatory microenvironment (3–7).

CTSS acts as a context-dependent, double-edged protease in pathophysiology. In some settings, CTSS amplifies inflammation, neuroimmune activation and tissue damage; in others, it may facilitate macrophage M2 polarization, matrix remodeling and wound healing. Therefore, the central question is not simply whether CTSS expression is increased, but when and where CTSS is active, which cells produce it, which substrates it cleaves, and whether its downstream effects are pathogenic or reparative. CTSS should be viewed as a context-dependent protease switch rather than a uniformly pathogenic enzyme. Although the highly selective CTSS inhibitor ASP1617 has been shown to prevent the progression of lupus-like glomerulonephritis in mice (8), recent clinical data in healthy subjects revealed a more complex pharmacodynamic profile. After repeated administration of high-dose ASP1617, CTSS activity gradually rebounded during treatment, exceeded baseline levels after the final dose, and was accompanied by a sustained increase in CTSS protein mass to approximately fivefold above baseline (9). These findings highlight the double-edged and context-dependent nature of CTSS biology and call for a careful reassessment of current strategies for CTSS inhibitor development.

As the largest immune organ in the human body, the skin is continuously exposed to diverse insults, and many dermatoses involve overlapping barrier, immune, neurogenic, oxidative, vascular, matrix-remodeling, and repair processes. CTSS may regulate these processes across immune, epithelial, endothelial, fibroblast, and sensory nerve-related compartments by cleaving substrates involved in antigen presentation, cytokine activation, receptor signaling, extracellular matrix turnover, itch, pain, and repair. This review summarizes the double-edged role of CTSS in inflammation and repair, highlights its relevance to dermatology, and discusses how this concept may guide more selective, compartment-aware, and stage-specific therapeutic strategies.

## 2 Molecular biology and regulation of CTSS

### 2.1 Gene localization, cellular sources, and zymogen maturation

The human CTSS gene is located on human chromosome 1q21 (10). CTSS is predominantly expressed in antigen-presenting cells, including dendritic cells, B cells, macrophages, and other mononuclear phagocytes, but it can also be produced by epithelial cells, endothelial cells, smooth muscle cells, neutrophils, and tumor cells (5, 6). In human blood, monocytes contain the highest levels of mature active CTSS, followed by neutrophils; CTSS activity in monocytes is approximately ten-fold higher than in neutrophils (11). This immune-cell-enriched expression profile provides a biological basis for CTSS involvement in inflammation, autoimmunity, antigen presentation, and tissue remodeling. **Figure 1** provides an overview of the intracellular regulation, maturation, trafficking, and lysosomal exocytosis of CTSS described in the following subsections.

**Figure 1:**
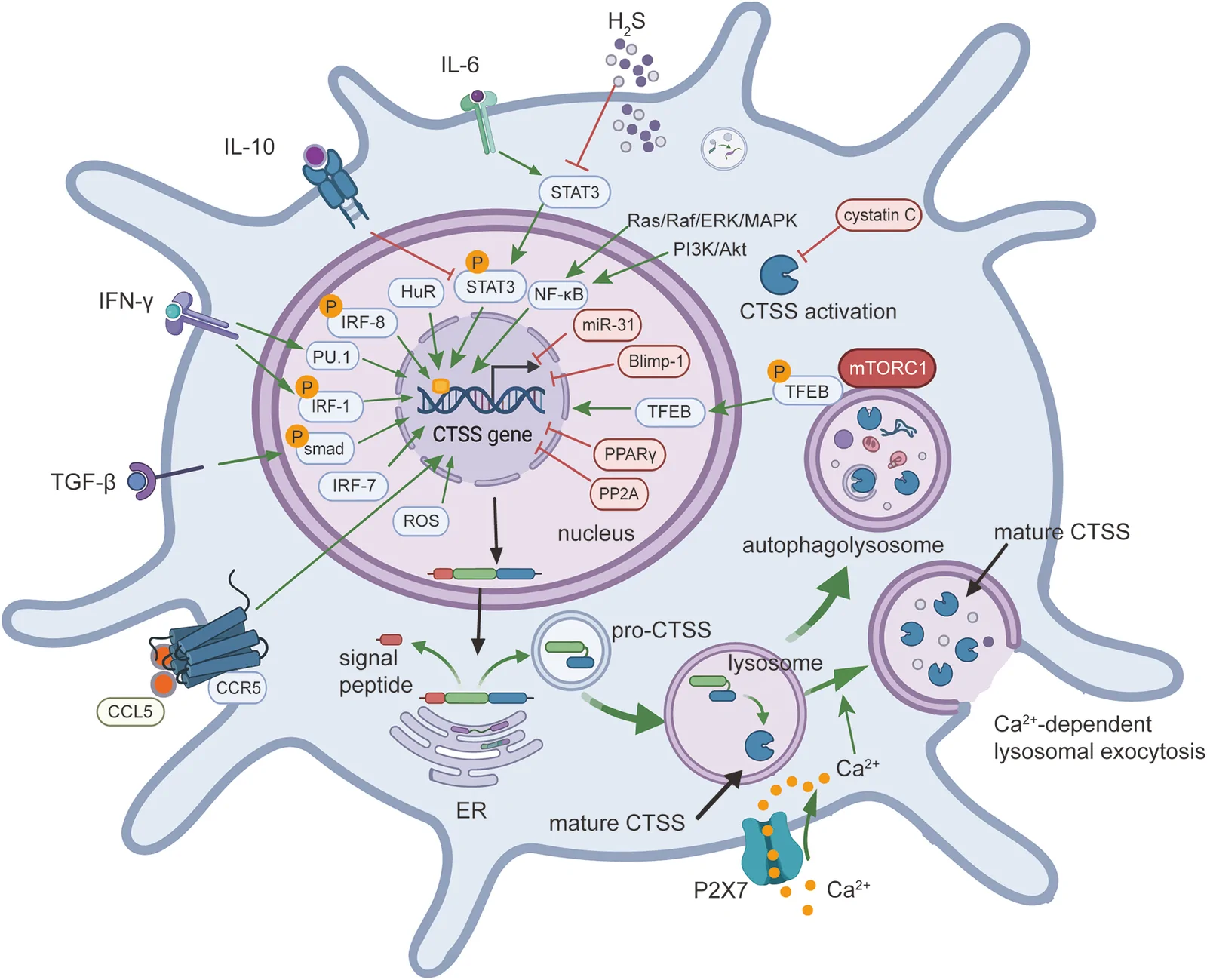
Intracellular regulation, maturation, and secretion of CTSS. CTSS expression and activity are regulated by inflammatory cytokines, transcription factors, autophagy-related pathways, endogenous inhibitors, and calcium-dependent lysosomal exocytosis. Pro-CTSS is processed into mature CTSS in lysosomes and may be released extracellularly, where it remains active under neutral or mildly acidic inflammatory conditions.

CTSS is synthesized as an inactive pre-proenzyme containing a signal peptide, propeptide, and mature catalytic domain. After signal peptide removal in the endoplasmic reticulum, the propeptide supports folding, endolysosomal targeting, and inhibition of premature activity (2, 10). Mature CTSS is a single-chain enzyme of approximately 217 amino acids and is mainly generated by autocatalytic processing under acidic conditions, which can be enhanced by glycosaminoglycans (10, 12). Alternative splicing or exon skipping may produce truncated CTSS forms with mitochondrial or nuclear localization, suggesting potential noncanonical roles in cell-cycle regulation or apoptosis (10, 13). CTSS can also be secreted through Ca²^+^-dependent lysosomal exocytosis (14, 15). In healthy individuals, CTSS activity in whole blood shows diurnal variation, with increased activity between 8:00 a.m. and 3:00 p.m., suggesting possible regulation by circadian endocrine factors such as cortisol (11).

### 2.2 pH stability and extracellular activity

CTSS is the only cysteine cathepsin which remains active at neutral or weakly alkaline pH, allowing it to function after secretion into inflamed or remodeled tissues (4, 16). Glycosaminoglycans and negatively charged surfaces further stabilize CTSS and promote pro-CTSS autocatalytic activation (4, 7, 12). Chondroitin sulfate A/E, dermatan sulfate, and dextran sulfate enhance CTSS activation at acidic pH, whereas dextran sulfate can also support its activation under neutral or slightly alkaline conditions (7, 10, 12). Several glycosaminoglycans abundant in the dermal matrix or basement membrane, including dermatan sulfate, hyaluronan, heparin, and chondroitin 6-sulfate, may modulate CTSS maturation and collagenolytic activity. In contrast, high concentrations of chondroitin 4-sulfate and heparan sulfate have been reported to suppress CTSS-mediated collagenolysis, highlighting the importance of glycosaminoglycans composition and sulfation patterns in regulating extracellular CTSS activity (7, 17). These observations are highly relevant to the skin, where dermal glycosaminoglycan composition changes during inflammation, aging, wound healing, scarring, photoaging, and fibrosis. The dermal ECM may therefore not only serve as a substrate for CTSS but also actively regulate CTSS maturation, stability, and collagenolytic activity. Such bidirectional interaction suggests that CTSS activity in skin is determined by a dynamic protease–matrix feedback loop rather than by enzyme expression alone.

### 2.3 Transcriptional and post-transcriptional regulation

CTSS expression, activity, and secretion are controlled by multiple regulatory layers in a cell- and context-dependent manner. CTSS expression is regulated by multiple molecules at both transcriptional and post-transcriptional levels. Environmental, psychological, infectious, and metabolic stressors, including cigarette smoke, nicotine, chronic stress, viral infection, and hyperglycemia, further enhance CTSS synthesis, secretion, or activity (18–22). These mechanisms position CTSS as a highly responsive protease node linking cytokine signaling, lysosomal trafficking, mRNA stability, redox regulation, and stress-induced inflammation.

Mature CTSS activity is tightly regulated by endogenous protease inhibitors, particularly the cystatin superfamily. Cystatin C is the best-characterized and one of the most potent endogenous CTSS inhibitors; it is broadly expressed, secreted extracellularly, and restrains excessive CTSS proteolytic activity (23). Other cystatin family members, such as cystatin F, as well as stefins, kininogens, thyropin-type inhibitors, and α2-macroglobulin, may also regulate CTSS in a cell- and compartment-dependent manner (6, 24, 25). ROS can promote cystatin C homodimer formation, weakening its inhibitory interaction with CTSS, whereas low intracellular ROS favors cystatin C-mediated inhibition (6, 26, 27). Notably, CTSS is relatively resistant to oxidants and can retain elastinolytic activity in oxidant-rich inflammatory environments, such as cigarette smoke exposure (10, 28). This feature may be relevant to oxidative stress-associated skin diseases, including psoriasis, AD, photoaging, chronic wounds, and melanoma.

At the transcriptional level, IFN-γ/IRF-1 (29), IRF7 (30), IRF8 (31), PU.1 (32, 33), and TFEB (21) can directly promote CTSS transcription, whereas Blimp-1 (34) and nuclear transglutaminase-2 (35) repress CTSS expression. The mTORC1–TFEB axis links lysosomal stress to CTSS synthesis and secretion, particularly under nicotine or inflammatory stimulation (21). STAT3-related regulation is cytokine dependent: IL-6–STAT3 signaling enhances cathepsin activity partly by reducing cystatin C (36), whereas IL-10–STAT3 signaling suppresses IFN-γ-induced CTSS-dependent MHC-II responses in macrophages (37). PI3K/Akt (38), Ras/Raf/ERK/MAPK (38), PPARγ-related signaling (39), and TGF-β/Smad pathways (40) have also been implicated in CTSS expression or CTSS-associated inflammatory, vascular, and fibrotic responses, although their effects vary across cell types and disease models. Post-transcriptionally, RNA editing can facilitate HuR binding to the CTSS 3′UTR and stabilize CTSS mRNA (41), whereas PP2A suppresses cigarette smoke-induced CTSS expression by promoting tristetraprolin-associated mRNA destabilization and inhibiting MAPK/ERK-dependent transcription (22). Expression of miR-31 negatively regulates CTSS production through the IRF-1 pathway (42). In addition, P2X7 receptor activation promotes CTSS release or upregulation through Ca²^+^-dependent mechanisms (43). Conversely, hydrogen sulfide can suppress the STAT3–CTSS axis, reducing CTSS-related neuroinflammation, elastin degradation, and vascular calcification (44, 45). The CCL5-CCR5 axis can induce retinal ganglion cell apoptosis induced by optic nerve injury by upregulating CTSS (46).

## 3 Intracellular and extracellular functions of CTSS

### 3.1 Antigen processing, immune surveillance and adaptive immune regulation

CTSS plays a canonical role in MHC-II-mediated antigen presentation. In antigen-presenting cells, invariant chain/CD74 guides MHC-II into endolysosomal compartments and prevents premature peptide loading. CTSS cleaves invariant chain to generate CLIP, which is removed by HLA-DM to permit antigen loading and CD4^+^ T-cell presentation (47–49). CTSS can also shape the self-antigen repertoire and immune tolerance. By processing autoantigens and destroying selected CD4^+^ T-cell epitopes, excessive CTSS activity may impair thymic negative selection and allow autoreactive T cells to escape (50). This provides a mechanistic link between CTSS overactivity, altered self-antigen presentation, and autoimmunity (**Figure 2**).

**Figure 2:**
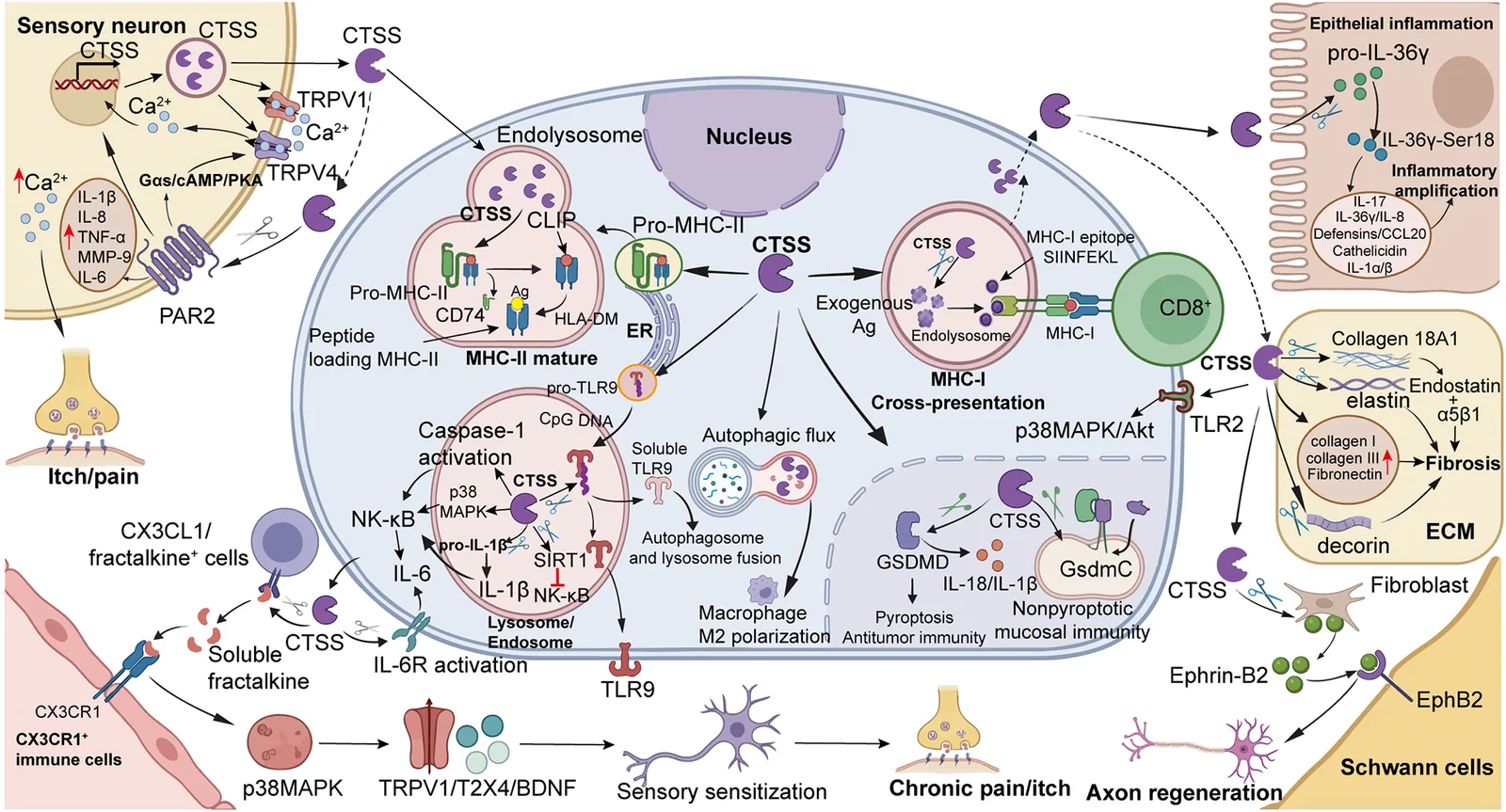
Major CTSS-mediated biological pathways. CTSS regulates antigen presentation, TLR/inflammasome signaling, cytokine activation, PAR2-mediated neuroimmune inflammation, fractalkine/CX3CL1 cleavage, extracellular matrix remodeling, fibrosis, axon regeneration, and tissue repair. These pathways explain how CTSS may connect immune activation, itch, pain, epithelial inflammation, and tissue remodeling.

CTSS can also contribute to MHC-I cross-presentation through a TAP-independent vacuolar pathway (51). CTSS-deficient antigen-presenting cells show impaired cross-presentation of particulate and cell-associated antigens, and recombinant CTSS can directly generate the MHC-I-presented SIINFEKL epitope from ovalbumin (10, 51, 52). These findings indicate that CTSS links endosomal proteolysis to CD8^+^ T-cell immunity and may influence antiviral, antitumor, and autoimmune responses in skin diseases.

### 3.2 Innate and epithelial inflammatory amplification

CTSS regulates Toll-like receptor signaling, especially TLR9. By cleaving TLR9 at the N- or C-terminus, CTSS can generate either functional TLR9 ectodomains or inhibitory soluble forms and influencing the balance between immune activation and suppression (53). CTSS is also linked to inflammasome-related inflammation. CTSS can compensate for other cathepsins and promote caspase-1 activation, leading to cleavage of pro-IL-1β into mature IL-1β (54). Additionally, CTSS can further amplify inflammation by degrading SIRT1 and thereby relieving SIRT1-mediated inhibition of NF-κB signaling (55). It can also cleave IL-6 receptor and promote IL-6 trans-signaling (56). These mechanisms position CTSS as an amplifier of inflammatory cytokine networks.

IL-36γ is a member of the IL-1 cytokine family and is highly expressed in epithelial tissues, including skin and lung. It is produced as an inactive precursor and requires proteolytic processing for activation. CTSS can cleave and activate IL-36γ, generating a highly active IL-36γ–Ser18 form that promotes epithelial inflammation (57). Active IL-36γ enhances expression of IL-36γ itself, IL-17 (58), IL-1α/β (59), and LL-37 (60), thereby amplifying epithelial inflammation and antimicrobial responses (10). In psoriasis, CTSS is a major activator of IL-36γ and is highly upregulated in psoriatic lesions (57).

Recent studies have expanded CTSS biology to the gasdermin family. In an antibody–indocyanine green near-infrared light-triggered tumor pyroptosis model, lysosomal damage causes CTSS to escape into the cytosol, where CTSS directly cleaves GSDMD, IL-18, and IL-1β, inducing caspase-1-independent pyroptosis and antitumor immunity (61). In contrast, CTSS directly cleaves GsdmC in intestinal epithelial cells, generating an N-terminal fragment that enters Rab7^+^ late endosomes and enhances anti-helminth type 2 immunity (62). These studies show that CTSS can act as a gasdermin-activating protease, but the biological consequence depends on the substrate, cell type, and subcellular compartment.

### 3.3 Neuroimmune signaling and immune-cell recruitment

Protease-activated receptor 2 (PAR2) is a G protein-coupled receptor involved in inflammation, pain, itch, angiogenesis, epithelial barrier regulation, and immune-cell activation. CTSS cleaves PAR2 near its N-terminus, exposing a tethered ligand distinct from the classical trypsin-generated tethered ligand (63). CTSS also activates PAR2 through biased agonism involving calcium mobilization, ERK1/2 activation, β-arrestin recruitment, receptor endocytosis, and TRPV4-related signaling (64).

Fractalkine, also known as CX3CL1, is a unique membrane-bound chemokine expressed by several structural and immune cells, including neurons, endothelial cells, vascular smooth muscle cells, monocytes, NK cells, and T cells. CTSS can cleave membrane-anchored fractalkine and release its soluble extracellular domain, thereby converting a cell-bound adhesion molecule into a diffusible chemotactic signal (10, 65). Membrane-bound fractalkine supports leukocyte adhesion to vascular endothelium through CX3CR1, while soluble fractalkine functions as a chemoattractant that promotes transmigration of immune cells into injured or inflamed tissues (10, 65). The CTSS/fractalkine/CX3CR1 axis is well established in pain and neuroinflammation. CTSS may convert membrane-bound chemokines into soluble leukocyte-recruiting mediators, thereby linking sensory nerves, endothelial cells, and immune-cell infiltration.

### 3.4 Inflammation resolution, extracellular matrix remodeling and repair

CTSS is required for autophagy and macrophage M2 polarization. In tumor-associated macrophages, CTSS deficiency leads to LC3-II accumulation, impaired autophagic flux, and reduced M2 polarization, suggesting that CTSS is necessary for autophagosome–lysosome fusion (66). CTSS is also important for anti-TNF-induced macrophage polarization toward an M2-like phenotype (67). CTSS inhibition can induce mitochondrial stress, ROS generation, mitochondrial apoptosis, and autophagosome accumulation in glioblastoma cells (68). Additionally, the lysosomal response triggered by autophagosome–lysosome fusion depends on TLR9 activation (69). Bioinformatic analyses have shown that CTSS is closely associated with macrophage phagocytosis and phagosome-related genes, and cellular studies further indicate that CTSS expression follows a similar pattern to these phagosomal markers (70). *In vivo*, Ctss deficiency or pharmacological inhibition reduces the expression of phagocytosis-related molecules, including gp91phox, p22phox, and p47phox, and decreases macrophage infiltration in chronic stress-related neointimal hyperplasia (18). Similarly, stress-induced upregulation of gp91phox and p22phox is reversed by Ctss knockout (71). Because these NADPH oxidase components are essential for LC3-associated phagocytosis (LAP), a key degradative process during efferocytosis (72, 73), these findings suggest that CTSS may participate in macrophage phagocytic and efferocytic regulation by modulating phagosome maturation and LAP-related oxidative machinery.

Cathepsins can cleave ECM, cell-surface proteins, cytokines, chemokines, and antimicrobial peptides, thereby regulating adhesion, migration, invasion, angiogenesis, and inflammatory signaling (4, 74). CTSS degrades structural ECM proteins such as collagen and elastin and can modify non-structural proteins, cytokines, chemokines, and receptor ectodomains (74, 75). It may also cleave collagen 18A1 to endostatin, leading to hepatic fibrosis (4, 10, 76). Previous reviews have highlighted the critical role of CTSS in the pathogenesis of pulmonary fibrotic diseases (6). Moreover, CTSS has been shown to be critical for type I and type III collagen fiber and fibronectin synthesis, suggesting a critical role in cutaneous fibrosis (30). These functions are particularly relevant to cutaneous wound repair. Macrophages must transition from pro-inflammatory phenotypes to reparative phenotypes, clear apoptotic cells, produce anti-inflammatory and pro-repair cytokines, and coordinate fibroblast and endothelial cell activity. CTSS may support these processes by maintaining lysosomal proteolysis, autophagic flux, efferocytosis-related degradation, and macrophage polarization. Thus, CTSS may contribute not only to inflammation initiation but also to inflammation resolution and tissue repair.

## 4 CTSS in dermatology

### 4.1 Psoriasis: CTSS links keratinocyte antigen presentation, IL-36γ inflammation, antimicrobial peptides, and barrier remodeling

Psoriasis is a chronic recurrent inflammatory skin disease involving keratinocyte hyperproliferation, innate immune activation, adaptive immune responses, angiogenesis, oxidative stress, antimicrobial peptide induction, and barrier dysfunction. Psoriasis is one of the skin diseases in which the link with CTSS has been most clearly demonstrated. CTSS may contribute to psoriasis through keratinocyte-associated antigen presentation, IL-36γ activation, antimicrobial peptide regulation, and ECM remodeling. Keratinocytes are not classical antigen-presenting cells, but under inflammatory conditions they may express MHC-II molecules. IFN-γ, a key cytokine in psoriasis, selectively upregulates CTSS activity in primary human keratinocytes at both mRNA and protein levels (77). CTSS upregulation correlates with MHC-II expression (77, 78) suggesting that CTSS may enhance antigen processing in keratinocytes and contribute to local T-cell activation in psoriatic lesions. A more disease-specific mechanism involves IL-36γ. IL-36γ is highly expressed in psoriatic epithelium and functions as a critical mediator of epithelial inflammation (57). CTSS is the major protease that processes inactive IL-36γ into the active IL-36γ–Ser18 form (57). This active cytokine induces keratinocyte inflammatory responses and can trigger epidermal hyperkeratosis and psoriasis-like changes in mouse models. CTSS is highly upregulated in psoriasis and is required for IL-36γ activation (57). Therefore, CTSS is not merely a marker of psoriasis inflammation but may function upstream of the IL-36 inflammatory loop (**Figure 3**).

**Figure 3:**
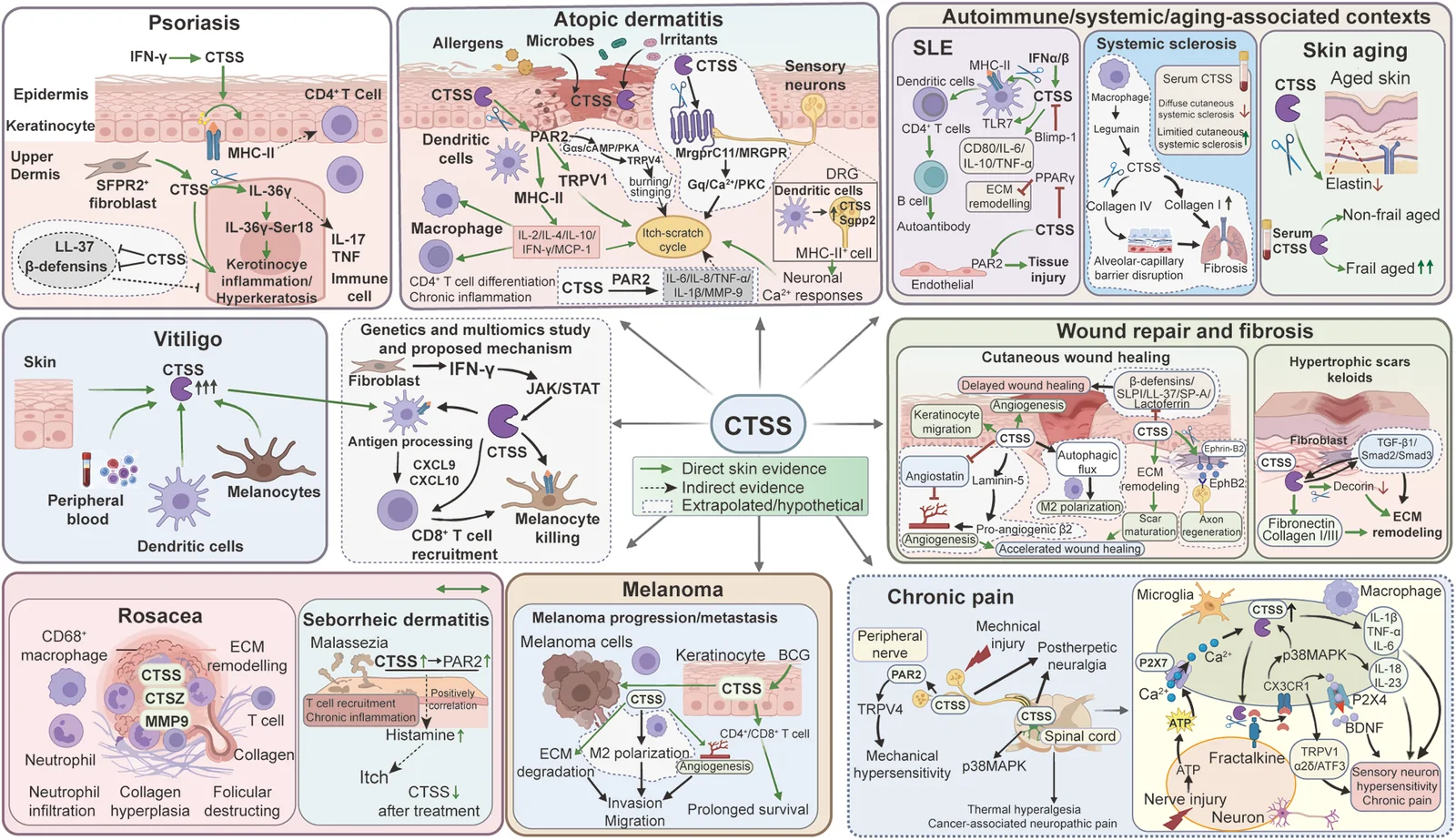
Context-dependent roles of CTSS in dermatological diseases. CTSS contributes to multiple skin-related diseases, including psoriasis, atopic dermatitis, vitiligo, rosacea, seborrheic dermatitis, melanoma, systemic lupus erythematosus, systemic sclerosis, skin aging, wound repair, keloids, and chronic pain. Its effects may be pathogenic or reparative depending on disease stage, cellular source, tissue compartment, and substrate specificity. Indirect non-cutaneous evidence is indicated by dashed boxes.

Single-cell and spatial transcriptomic analyses identified inflammatory SFRP2^+^ fibroblasts as key amplifiers of psoriatic inflammation (79). These fibroblasts, mainly localized in the upper dermis and dermal papillae, express CTSS, which may activate keratinocyte-derived IL-36γ and reinforce an IL-36-dependent inflammatory loop driven by IL-17A and TNF in the supraspinous epidermis (79). These findings expand the role of CTSS in psoriasis beyond immune-cell antigen processing and suggest that fibroblast-derived CTSS may contribute to epithelial–stromal inflammatory crosstalk.

CTSS may also interact with antimicrobial peptide networks in psoriasis. LL-37 and β-defensins are strongly implicated in psoriasis pathogenesis, where they can act as antimicrobial peptides, immune modulators, and autoantigen-related mediators. Excessive CTSS may degrade LL-37 and β-defensins (80, 81), potentially reshaping antimicrobial defense and inflammatory signaling in cystic fibrosis. Whether CTSS-mediated cleavage of LL-37 modifies its ability to form nucleic acid complexes and activate plasmacytoid dendritic cells in psoriasis remains unknown but is a valuable research direction.

### 4.2 AD: CTSS connects barrier dysfunction, dendritic-cell activation, cytokine release, and itch

AD is characterized by chronic relapsing eczema, epidermal barrier dysfunction, immune dysregulation, microbial dysbiosis, and intense pruritus. CTSS overexpression induces spontaneous chronic AD-like skin disease in transgenic mice (82). Intradermal injection of recombinant human CTSS can directly activate sensory neurons and induces scratching behavior in mice, and this effect can be blocked by CTSS inhibitors or PAR2 antagonists (63). CTSS-induced itch is partially reduced in TRPV1-deficient mice, suggesting crosstalk between CTSS–PAR2 signaling and sensory ion channels (63). In AD-like mice, CTSS overexpression upregulates PAR2 in dendritic cells, enhances CD4^+^ T-cell differentiation, and is associated with MHC-II expression, thereby promoting scratching behavior, Th1-related cytokine production, and chronic AD-like inflammation (82). CTSS/PAR2 signaling may further enhance cytokine and chemokine release, including IL-2, IL-4, IL-10, IFN-γ, and MCP-1, linking protease activity to dendritic-cell activation, T-cell responses, and pruritic inflammation (5, 82). In human corneal epithelial cells, CTSS upregulated IL-6, IL-8, TNF-α, IL-1β, MMP-9, and PAR2 partly through PAR2-dependent signaling, supporting a broader role for the CTSS–PAR2 axis in epithelial inflammation (83). CTSS can cleave PAR2 at a noncanonical site and act as a biased PAR2 agonist, preferentially engaging Gαs–cAMP–PKA signaling and activating TRPV4 rather than inducing classical trypsin-like Gq/β-arrestin signaling (64). Although this biased PAR2–TRPV4 mechanism was primarily defined in inflammatory pain models (64), it provides a plausible framework for understanding CTSS-associated cutaneous dysesthesia, including burning, and stinging sensations in AD and related inflammatory dermatoses. CTSS-evoked non-histaminergic itch is mediated predominantly by Mas-related G protein-coupled receptors (MRGPRs). In sensory neurons, CTSS cleaves the extracellular N-terminus of MrgprC11 between N16 and E17, activating Gq-dependent Ca²^+^ signaling and PKC phosphorylation without generating a functional tethered ligand (84). *In vivo*, CTSS-induced scratching is markedly reduced in MrgprC11/MRGPR cluster-deficient mice but not in PAR2-deficient mice, indicating that MRGPRs, rather than PAR2, are essential for CTSS-evoked itch (84). A recent study linked CTSS to dorsal root ganglion (DRG)-associated neuroimmune sensitization in allergic contact dermatitis. In a toluene diisocyanate-induced mouse model of allergic contact dermatitis, mast cells and MHC-II^+^ dendritic cell-like cells increased in the DRG, with sorted MHC-II^+^ cells showing upregulated Ctss and Sgpp2 and enriched immune-response and antigen-presentation pathways (85). CTSS inhibition reduced DRG neuronal calcium responses to histamine, AITC, and capsaicin in dendritic cell–neuron co-cultures, suggesting that CTSS promotes peripheral itch sensitization through DRG neuroimmune interactions (85).

Collectively, these findings support a model in which CTSS participates in the itch–scratch–barrier disruption cycle. Barrier injury permits allergens, microbes, and irritants to penetrate the epidermis, leading to epithelial cytokine release and immune-cell activation. CTSS produced by keratinocytes, dendritic cells, macrophages, or infiltrating inflammatory cells may activate PAR2 on dendritic cells and sensory neurons, thereby amplifying local inflammation and itch. Scratching further damages the epidermal barrier, promotes additional inflammatory-cell recruitment and protease release, and establishes a self-perpetuating loop of barrier disruption, neuroimmune activation, and chronic pruritic inflammation.

### 4.3 Vitiligo: CTSS links antigen presentation to melanocyte destruction

Integrative genetics and multiomics study recently identified CTSS as a potential causal and therapeutically relevant gene in vitiligo. The study showed that CTSS was associated with vitiligo across both blood and skin datasets and was differentially expressed in peripheral blood and lesional skin (86). CTSS was highly expressed in dendritic cells and was also detected in lesional melanocytes, supporting its potential role in antigen processing and presentation. Importantly, bioinformatic analysis suggested that STAT1 and STAT3 may bind to regulatory regions of CTSS, with enrichment of the JAK/STAT pathway observed in the high-CTSS expression group (86). The central mechanisms of vitiligo include IFN-γ/JAK/STAT activation, CXCL9/CXCL10-mediated T-cell recruitment, CD8^+^ T-cell-mediated melanocyte destruction, and melanocyte antigen presentation (86). CTSS may contribute to these processes by enhancing antigen processing or altering MHC-associated peptide repertoires, thereby influencing melanocyte–immune cell interactions. However, current evidence is mainly based on multiomics association and bioinformatic inference. Functional validation in lesional skin, melanocyte models, and immune co-culture systems is still required.

### 4.4 Rosacea: CTSS as a link between immune activation and ECM remodeling

Granulomatous rosacea provides evidence linking CTSS to skin granulomatous inflammation and tissue remodeling. In a comparative study of granulomatous and non-granulomatous rosacea, granulomatous lesions showed greater neutrophil, T-cell, and CD68^+^ macrophage infiltration, along with collagen hyperplasia and follicular destruction (87). RNA-seq revealed 420 differentially expressed genes enriched in neutrophil activation/degranulation, adaptive immunity, and T-cell activation (87). Among granuloma-associated candidates, CTSS, CTSZ, and MMP9 were significantly upregulated and positively associated with neutrophil infiltration and collagen hyperplasia (87). These findings suggest that CTSS may contribute to granulomatous rosacea by linking antigen-processing macrophage-rich inflammation, neutrophil activation and ECM remodeling.

### 4.5 Seborrheic dermatitis: CTSS as a pruritus-associated biomarker

Scalp seborrheic dermatitis and dandruff are common inflammatory scalp disorders characterized by *Malassezia* colonization, impaired barrier function, erythema, scaling, and pruritus. Histamine is widely used as a biomarker for itch evaluation and correlates with disease severity. PAR2 and CTSS are highly expressed in scalp seborrheic dermatitis, and their expression correlates with increased histamine levels (88). CTSS may therefore serve as a pruritus-associated biomarker and may help evaluate treatment response in dandruff and seborrheic dermatitis. A metabolomic and fungal microbiome study of mild-to-moderate scalp seborrheic dermatitis showed that a 2-week intensive anti-dandruff shampoo regimen significantly reduced scalp CTSS levels by 64.6%, together with decreases in IL-8, histamine, arachidonic acid, linoleic acid, oxylipins, and tryptophan-derived indole metabolites (89). During the maintenance phase, CTSS remained low in subjects who continued weekly use of the anti-seborrheic dermatitis shampoo, whereas CTSS rebounded in the control group using only a neutral shampoo (89). These findings support CTSS as a clinically responsive biomarker of scalp pruritus and inflammation and place CTSS within a broader Malassezia–lipid metabolism–inflammatory mediator network in seborrheic dermatitis.

### 4.6 Systemic lupus erythematosus and cutaneous lupus relevance

Systemic lupus erythematosus is a systemic autoimmune disease with frequent cutaneous manifestations. CTSS has been implicated in lupus pathogenesis through MHC-II antigen presentation, TLR signaling, interferon responses, autoantibody production, PAR2-mediated tissue injury, and immune tolerance disruption. CTSS-overexpressing transgenic mice spontaneously develop typical SLE-like symptoms after aging, accompanied by increased TLR7, autoantibodies, and IFN-α expression (90). IFN-α/β significantly upregulates CTSS expression in human endothelial cells, while influenza vaccination-induced type I IFN activation also elevates CTSS expression in both SLE patients and healthy subjects (91). Intravenous CTSS induces severe glomerular endothelial injury and proteinuria, both of which can be blocked by CTSS or PAR2 inhibitors. *In vitro*, CTSS induces endothelial activation and injury through PAR2 (92). These findings show that CTSS can promote both systemic autoimmunity and peripheral tissue injury. In lupus mouse models, CTSS inhibition reduces plasma IL-10 and TNF-α, disrupts activation of CD4^+^ T cells and autoreactive B cells, attenuates IgG autoantibody production, suppresses renal pathology, and improves pulmonary symptoms (93). And CTSS inhibition has been shown to upregulate peroxisome proliferator-activated receptor-γ and attenuates SLE-associated pulmonary arterial remodeling and right ventricular hypertrophy in experimental models (94). CD8^+^ T cell number and activation are not substantially affected, indicating that CTSS inhibition primarily acts by interfering with MHC-II-dependent CD4^+^ T cell and B cell priming (93). In SLE patient-derived immune cells, CTSS inhibition reduces MHC II and CD80 expression, suppresses IL-6, TNF-α, and IL-10 secretion, and attenuates anti-C1q-induced proinflammatory macrophage activation, supporting CTSS as a potential therapeutic target in SLE (95). Blimp-1 suppresses CTSS expression in dendritic cells, whereas Blimp-1 deficiency increases CTSS, alters the T follicular helper cell repertoire, and contributes to lupus-like autoimmunity (34). Excessive CTSS can also destroy T-cell epitopes of self-antigens, impair negative selection, and skew the T-cell receptor repertoire toward self-reactivity (10, 50). This immune-tolerance mechanism may be highly relevant to cutaneous lupus, where interferon signaling, antigen presentation, endothelial injury, immune-complex inflammation, and tissue remodeling contribute to skin lesions.

### 4.7 Systemic sclerosis

Systemic sclerosis is characterized by vasculopathy, immune activation, fibrosis, and skin thickening. Interestingly, CTSS is not always increased in fibrotic disease. Patients with diffuse cutaneous systemic sclerosis show significantly lower serum CTSS levels than patients with limited cutaneous disease and healthy controls (96). Reduced serum CTSS levels are associated with Raynaud’s phenomenon and interstitial lung disease. Serum CTSS negatively correlates with Krebs von den Lungen-6 and surfactant protein D, is not correlated with total skin thickness score or predicted diffusing capacity for carbon monoxide, and positively correlates with predicted forced vital capacity (96). Macrophage-derived legumain activates CTSS, thereby mediating basement membrane collagen IV degradation and disruption of the alveolar–capillary barrier, while also promoting collagen I deposition; together, these effects foster pulmonary fibrosis (97). So CTSS may be involved in systemic sclerosis in a more complex manner. In some contexts, excessive CTSS drives inflammation and fibrosis; in others, reduced CTSS may reflect impaired matrix turnover, altered immune regulation, or defective protective remodeling.

### 4.8 Skin aging and elastin remodeling

Skin aging is associated with epidermal thinning, dermal ECM disorganization, elastin fragmentation, collagen alteration, reduced repair capacity, and chronic low-grade inflammation. Cysteine cathepsins are major matrix-degrading proteases, CTSK, CTSS, and CTSV are involved in elastin degradation, and CTSS expression is significantly reduced in aged human skin, especially in the epidermal compartment (98). CTSS may therefore serve as a marker of age-related changes in epidermal protease networks. However, in a cross-sectional study of 403 home-dwelling individuals aged 70 years or older, frailty index scores were positively associated with CTSS (99). CTSS levels were significantly higher in frail than in non-frail participants and remained independently associated with frailty index after adjustment for age, sex, BMI, and smoking (99). Frailty in female participants was also associated with CIBERSORT-predicted monocyte signatures, and monocyte-related genes showed associations with inflammatory biomarkers, including CTSS (99). These findings suggest that CTSS may serve as a biomarker of frailty-related low-grade inflammation, although causal relationships require longitudinal validation.

The apparently contradictory changes in CTSS expression in older patients may reflect the broad and context-dependent functions of this protease. Although reduced CTSS expression in aging skin seems paradoxical given its elastolytic activity, skin aging should not be viewed simply as a process of excessive matrix degradation. Rather, it involves impaired ECM turnover, defective tissue repair, altered immune surveillance, delayed barrier recovery, and disruption of the protease–inhibitor balance. Therefore, reduced CTSS expression in aged epidermis may indicate weakened immune-proteolytic activity and insufficient homeostatic remodeling, rather than a purely protective reduction in matrix degradation.

### 4.9 Cutaneous wound healing

Cutaneous wound healing requires coordinated hemostasis, inflammation, re-epithelialization, angiogenesis, granulation tissue formation, ECM deposition, remodeling, and restoration of barrier function. CTSS may support wound repair through multiple mechanisms. CTSS remodels ECM proteins, allowing migration of keratinocytes, fibroblasts, endothelial cells, and immune cells. CTSS promotes angiogenesis. Ctss-deficient mice show markedly impaired microvessel growth during skin wound healing, with microvessel numbers reduced by approximately 80% (100). CTSS can degrade angiogenesis inhibitors such as angiostatin and endostatin and generate pro-angiogenic β2 fragments from laminin-5, thereby promoting microvascular formation in mouse pancreatic tumor (101). CTSS supports autophagic flux in anti-TNF-induced macrophages and promotes M2 polarization in tumor-associated macrophages, which are important for inflammatory resolution and repair (66, 67). M2 macrophage-derived CTSS promotes mouse axon regeneration through a Schwann cell-fibroblast signaling relay involving EphB2 and Ephrin-B2 combination (102). CTSS also stimulates proliferation and migration of human periodontal ligament cells, supporting a broader role in wound closure and tissue remodeling (103).

CTSS may also influence wound inflammation through inactivating protective antimicrobial and anti-inflammatory mediators. Secretory leukoprotease inhibitor (SLPI) involved in epithelial defense and wound repair, can inhibit neutrophil elastase and has anti-inflammatory and antimicrobial activities. CTSS cleaves and inactivates SLPI, potentially worsening barrier injury and prolonging pulmonary inflammation (104) (105). CTSS also cleaves surfactant protein-A, a protective innate immune molecule in the lung, increasing susceptibility to inflammation and recurrent infection (105). It degrades human β-defensins 2 and 3, impairing antimicrobial defense (80). CTSS cleaves lactoferrin and LL-37, reducing their activity against bacteria such as *Escherichia coli*, *Staphylococcus aureus*, and *Pseudomonas aeruginosa* (81, 106). Although much of this evidence comes from cystic fibrosis and lung disease, the implications are relevant to skin because LL-37, β-defensins, and SLPI are key antimicrobial and immunomodulatory components of epidermal barrier defense. LL-37 and β-defensins are strongly implicated in psoriasis, AD, acne, wound repair, and infection-prone dermatoses, whereas SLPI contributes mainly to cutaneous antimicrobial protection, protease control, inflammation resolution, and wound healing, with emerging evidence for a protective role in psoriasis-like skin inflammation (107–110). This mechanism suggests a paradoxical role for CTSS in barrier immunity. Physiological CTSS supports antigen processing and host defense, whereas excessive extracellular CTSS may degrade antimicrobial peptides and anti-inflammatory inhibitors, thereby weakening barrier protection and sustaining inflammation.

### 4.10 Hypertrophic scarring and keloid formation

Pathological scarring results from dysregulated wound repair characterized by excessive fibroblast activation, collagen deposition, altered ECM turnover, persistent inflammation, angiogenesis, and mechanical signaling. CTSS contributes to scar formation by promoting expression of collagen I/III and fibronectin after injury (30). CTSS overexpression has been proposed as a pivotal factor in keloid formation, and CTSS inhibition reduces scar size in mouse skin incision models (30). Decorin provides another potential mechanistic link between CTSS and cutaneous fibrosis. Decorin is an abundant ECM proteoglycan and regulates collagen fibrillogenesis and tissue homeostasis. Decorin knockout mice exhibit abnormal collagen fibril formation, enhanced collagen degradation, fragile skin, and reduced tensile strength (111). CTSS-mediated decorin degradation is elevated in fibrotic lung disorders (111). By analogy, CTSS may participate in cutaneous fibrosis and post-wound remodeling by cleaving decorin, disrupting ECM homeostasis, and altering collagen architecture.

Additionally, studies of fibrosis in organs beyond the skin may provide new insights into cutaneous remodeling. TGF-β1/Smad2/Smad3 signaling can promote fibroblast dedifferentiation through CTSS and increase ECM-associated proteins in cardiac fibrosis, whereas TGF-β/Smad4 inhibition may increase CTSS-dependent ECM remodeling and protect against aneurysm formation in other contexts (112–114). CTSS may regulate renal fibrosis through the TGF-β/Smad signaling pathway in hydronephrosis models, supporting a tissue- and fibrosis-context-dependent role of CTSS in ECM remodeling (115). These apparently divergent observations suggest that the functional outcome of TGF-β/Smad–CTSS crosstalk depends on disease context, tissue compartment, and stage-specific regulation.

### 4.11 Cutaneous melanoma and tumor microenvironment remodeling

CTSS has complex and context-dependent roles in cancer biology. It can promote tumor progression by degrading ECM, enhancing angiogenesis, facilitating invasion and migration, supporting tumor-associated macrophage function, and modifying immune signaling. In several malignancies, including uveal melanoma and glioblastoma, elevated CTSS expression has been associated with angiogenic and anti-apoptotic pathways, tumor dissemination, and poor prognosis (116). CTSS can also regulate BRCA1 stability by promoting ubiquitin-mediated degradation, thereby impairing DNA damage repair and contributing to breast cancers (117). Across cancers, CTSS expression is closely associated with tumor microenvironment features, immune infiltration, and prognosis (118–120).

A recent two-sample Mendelian randomization and bioinformatics study based on large-scale genomic cohort data involving approximately 500,000 individuals further highlighted the dual role of CTSS in cutaneous melanoma (CM) (116). CTSS expression is significantly increased in both primary and metastatic CM compared with normal skin. High CTSS expression positively correlates with tumor mutation burden, tumor microenvironment activity, immune-cell infiltration, immune checkpoint expression, and predicted immunotherapy response s (116). CTSS promotes M2 polarization of tumor-associated macrophages, thereby potentially fostering an immunosuppressive niche that supports tumorigenesis (66, 67). In contrast, high CTSS expression is accompanied by increased CD8^+^ and CD4^+^ T cell infiltration, prolonged survival, and improved immunotherapy responsiveness (116). Consistent with this immune-promoting function, BCG lysate or BCG hydrogel has been reported to upregulate CTSS-mediated antigen processing and presentation, suppress melanoma metastasis, and prolong survival (121). The prognostic importance of immune infiltration is further supported by evidence that tumor-infiltrating lymphocyte grade is independently associated with melanoma-specific survival (122). These apparently divergent findings may partly reflect the dual role of CTSS in inflammation and immunity and may help explain why high CTSS expression can be associated with both tumor development and favorable immune-related prognosis in CM.

Additional cancer-related mechanisms may also be relevant to melanoma. Tumor-cell- or stromal-cell-derived CTSS may facilitate invasion and migration through ECM degradation and intracellular signaling, whereas tumor-associated macrophage derived CTSS may promote M2 polarization and a tumor-supportive microenvironment (66). In contrast, dendritic cell- or tumor-cell-associated CTSS may enhance antigen processing and T cell activation. Therefore, CTSS should not be viewed simply as a tumor-promoting protease. Its net effect in melanoma likely depends on whether CTSS activity predominates in tumor cells, tumor-associated macrophages, dendritic cells, or T-cell-rich immune niches. Simple CTSS blockade may reduce invasion and angiogenesis but could also impair antigen presentation and antitumor immunity. Future melanoma therapies targeting CTSS should therefore be mechanism-based, cell-specific, and context-specific rather than relying on broad CTSS inhibition alone.

### 4.12 Chronic pain

CTSS has emerged as a neuroimmune regulator and a potential therapeutic target for chronic pain. In preclinical models of neuropathic and inflammatory pain, CTSS is upregulated in dorsal root ganglia, injured nerves, and the spinal dorsal horn, where it is mainly derived from infiltrating macrophages or activated microglia (123, 124). In classical neuropathic pain models, nerve injury triggers ATP release, which activates P2X7 receptors on microglia or macrophages and promotes CTSS secretion. Extracellular CTSS then cleaves neuronal membrane-bound fractalkine to generate soluble fractalkine, which activates CX3CR1 on microglia or macrophages and induces the release of TNF-α, IL-1β, and other inflammatory mediators, thereby sustaining central sensitization and chronic pain (124–126). This CTSS/fractalkine/CX3CR1 axis also promotes the release of pronociceptive cytokines, contributing to central sensitization and chronic pain (127–129). In addition, peripheral nerve injury–induced activation of this pathway upregulates neuro-sensitizing molecules, including TRPV1, P2X4, brain-derived neurotrophic factor (BDNF), α2δ calcium channels, and ATF3 (124–126, 130–132). These downstream mediators may promote sensory neuron sensitization and sustain chronic pain or itch-like neuroimmune signaling. Recent work further suggests that the biological effect of fractalkine depends on proteolytic processing and ligand concentration. Soluble full-length fractalkine generated by ADAM10/17-like cleavage and chemokine domain-only fractalkine, which resembles shorter fragments potentially generated by CTSS, are not functionally equivalent (133). Low concentrations of fractalkine tend to suppress microglial TNF-α release, whereas high concentrations may lose anti-inflammatory activity or even promote inflammation (133). Therefore, the CTSS-fractalkine axis should not be interpreted simply as “CTSS releases fractalkine.” Instead, fragment length, local ligand concentration, receptor bias, and cellular activation state must be considered.

In the periphery, CTSS can also cleave and activate PAR2, leading to TRPV4 activation in sensory neurons and enhanced nociceptive signaling (64, 134). CTSS can proteolytically cleave the extracellular N-terminal domain of PAR2, thereby removing the N-terminal sequence and activating PAR2-dependent signaling, and contributing to cancer-associated mechanical allodynia and thermal hyperalgesia (135). Selective, orally bioavailable, non-peptidic CTSS inhibitors were reported to reverse established mechanical hyperalgesia in a rat model of chronic neuropathic pain (136). Given its roles in neuroimmune inflammation, protease-activated receptor signaling, and peripheral sensitization, CTSS may serve as a promising mechanistic and therapeutic target for chronic pain in skin diseases, particularly postherpetic neuralgia.

### 4.13 Emerging relevance to skin disease

Evidence from mouse abdominal aortic aneurysm models further supports the role of CTSS in ECM remodeling (137). In vascular smooth muscle cells, BAF60a-dependent chromatin remodeling facilitates NF-κB/BRG1-mediated transcriptional activation of CTSS (137). Loss of BAF60a markedly decreased CTSS expression and secretion, accompanied by reduced elastin fragmentation and ECM degradation in aneurysmal lesions (137). Given the broad expression pattern of BAF60a across multiple tissues and cell types, this epigenetic regulatory mechanism may not be restricted to the vascular wall. It can be speculated that BAF60a may also have biological relevance in skin, where ECM turnover, inflammatory activation, and tissue remodeling are central to wound repair, fibrosis, photoaging, and inflammatory dermatoses. Therefore, the BAF60a-NF-κB/BRG1-CTSS axis may represent a potential upstream mechanism linking chromatin remodeling to CTSS-mediated matrix proteolysis in cutaneous pathology. The context-dependent roles of CTSS across dermatological diseases and the corresponding levels of evidence are summarized in **Table 1**.

**Table 1:** Context-dependent roles of CTSS in dermatological diseases.

Disease	Principal CTSS sources	Known or proposed substrate/pathway	Mechanism/phenotype	Direction	Evidence level	Key references
Psoriasis	Keratinocytes; SFRP2+ inflammatory fibroblasts	Invariant chain/MHC-II; IL-36γ;	IFN-γ upregulates keratinocyte CTSS and may enhance local antigen processing. CTSS processes inactive IL-36γ into the active IL-36γ–Ser18 form and may amplify IL-36/IL-17/TNF epithelial-stromal inflammation.	Pathogenic	Human lesional skin; human keratinocytes in primary culture; Hacat cells; Cutaneous animal model; *In vitro* biochemical evidence	(57, 77–79)
Atopic dermatitis and allergic pruritic inflammation	Dendritic cells; keratinocytes; macrophages/inflammatory cells; DRG-associated MHC-II+ cells	PAR2; MrgprC11/MRGPRs; MHC-II; TRPV1/TRPV4-associated sensory signaling	CTSS overexpression produces chronic AD-like dermatitis and promotes dendritic-cell activation, T-cell responses and scratching. CTSS activates non-histaminergic itch through MrgprC11.	Pathogenic	Cutaneous animal model; *In vitro* biochemical evidence	(63, 82–85)
Vitiligo	Dendritic cells; lesional melanocytes; peripheral blood	JAK/STAT-associated CTSS regulation; antigen processing and MHC-associated peptide presentation (proposed)	CTSS is associated with vitiligo across blood and skin datasets and may facilitate melanocyte antigen presentation and cytotoxic T-cell responses. Causality and relevant substrates in skin remain unverified.	Pathogenic	Human lesional skin; *In vitro* biochemical evidence	(86)
Granulomatous rosacea	Skin lesions	Antigen-processing pathways; neutrophil activation; ECM remodeling (proposed)	CTSS is upregulated in granulomatous lesions and correlates with neutrophil infiltration and collagen hyperplasia, suggesting a link between granulomatous inflammation and matrix remodeling; functional causality has not been established.	Pathogenic	Human lesional skin; *In vitro* biochemical evidence	(87)
Scalp seborrheic dermatitis	Skin lesions	PAR2-associated pruritic signaling (proposed); inflammatory and lipid-metabolite networks	Scalp CTSS correlates with histamine and disease activity and decreases with effective anti-dandruff treatment, supporting its role as a treatment-responsive pruritus/inflammation biomarker; a causal mechanism is unconfirmed.	Pathogenic	Human lesional skin; *In vitro* biochemical evidence	(88, 89)
Systemic lupus erythematosus	Dendritic cells; other antigen-presenting cells; endothelial cells	Invariant chain/MHC-II; TLR7/IFN-alpha; PAR2; self-antigen processing and immune tolerance	Excess CTSS promotes MHC-II-dependent CD4+ T-cell/B-cell priming, autoantibody production and lupus-like disease, and may alter negative selection. Relevance to cutaneous lupus is mechanistically plausible but not directly demonstrated in skin lesional.	Pathogenic	Animal model/extrapolated evidence; *In vitro* biochemical evidence	(34, 90–95)
Systemic sclerosis-associated fibrotic disease	Serum of peripheral blood	Matrix turnover and immune regulation (proposed)	Lower serum CTSS is associated with diffuse disease features and interstitial lung disease, but not with skin thickness; direct dermal evidence is lacking.	Uncertain	Non-cutaneous model/extrapolated evidence (human serum)	(96)
Skin aging	Skin lesions; peripheral blood	Elastin degradation; frailty-associated pathways (proposed)	CTSS expression is reduced in aged human epidermis, potentially indicating impaired homeostatic remodeling, whereas circulating CTSS is increased in frailty-related inflammation. Tissue and circulating findings point in different directions.	Context-dependent	Human skin lesional; Extrapolated evidence (human peripheral blood biomarkers)	(98, 99)
Cutaneous wound healing	Skin lesions	Angiostatin	Ctss-deficient mice show markedly impaired microvessel growth.	Protective	Cutaneous animal model	(100)
Hypertrophic scarring and keloid formation	Fibroblasts and wound-associated stromal	Type I/III collagen; fibronectin; decorin	CTSS promotes collagen/fibronectin accumulation after injury, and its inhibition reduces scar size in a mouse incision model. CTSS-mediated decorin degradation may disrupt ECM homeostasis, thereby weakening skin integrity and potentially promoting cutaneous fibrosis.	Context-dependent	Cutaneous animal model; human fibroblasts	(30, 111)
Cutaneous melanoma	Human lesional skin	ECM degradation; angiogenic factors; macrophage M2 polarization; antigen processing and presentation; immune-checkpoint-associated pathways	Elevated CTSS expression has been associated with angiogenic and anti-apoptotic pathways, tumor dissemination, and poor prognosis.	Pathogenic	Human lesional skin	(116)

## 5 Therapeutic targeting of CTSS in skin diseases: opportunities, limitations, and future directions

### 5.1 CTSS inhibition: therapeutic potential and clinical challenges

Several CTSS inhibitors have entered clinical or preclinical development, but their clinical translation remains limited. In psoriasis, the selective CTSS inhibitor RO5459072 was tested in an open-label phase 2a study of 30 adults with moderate-to-severe psoriasis at 100 mg twice daily for 12 weeks (138). Although CTSS can activate IL-36γ into the proinflammatory IL-36γ–Ser18 fragment, RO5459072 failed to achieve PASI75 or improve secondary endpoints despite adequate drug exposure (138). The drug was generally well tolerated, but one treatment-related serious skin infection occurred (138). These findings suggest that selective inhibition of CTSS-mediated IL-36γ activation is insufficient to control psoriatic inflammation. One possible explanation for the disconnect between CTSS target engagement and clinical efficacy is protease redundancy. The time- and concentration-dependent release of elastase, cathepsin G, and other IL-36γ-processing proteases from infiltrating neutrophils may provide compensatory processing capacity and sustain IL-36-driven inflammation despite selective CTSS inhibition (138). Thus, selective CTSS inhibition may reduce one route of IL-36γ activation without fully suppressing the pathway *in vivo*.

Similar limitations have been observed with other CTSS inhibitors. VBY-891, RWJ-445380, and CRA-028129 have been tested in psoriasis-related programs, but none has been approved (10, 139). In primary Sjögren’s syndrome, RO5459072 achieved pharmacodynamic target engagement but failed to improve clinical, glandular, or quality-of-life outcomes compared with placebo, despite mild immunological effects (140). These findings indicate that target engagement does not necessarily translate into clinical efficacy in complex inflammatory diseases. ASP1617 further revealed challenges of long-term CTSS inhibition. Although it rapidly inhibited CTSS activity and induced Lip10 accumulation in B cells, repeated dosing increased CTSS mass and caused rebound CTSS activity above baseline, with rash and throat tightness at higher doses (9). These findings suggest that sustained CTSS inhibition may induce compensatory CTSS upregulation or broader protease-network rebound, thereby limiting efficacy and potentially increasing the risks associated with extracellular CTSS activity. Although topical delivery may reduce systemic exposure, it cannot be assumed to prevent the CTSS accumulation and activity rebound observed with ASP1617, because similar compensatory responses may occur locally. Moreover, rapid epidermal clearance, limited skin penetration, and inadequate access to intracellular or extracellular CTSS compartments may result in incomplete or transient inhibition, highlighting the need for controlled-release, compartment-directed formulations and parallel monitoring of local and systemic CTSS activity and mass. **Table 2** summarizes CTSS inhibitors under preclinical and clinical development.

**Table 2:** CTSS-targeted agents in preclinical and clinical development.

Agent	Modality	Target indication	Development stage	Key pharmacodynamic outcomes/failure reasons	References
RO5459072 (petesicatib)	Oral small-molecule inhibitor	Psoriasis; Sjogren’s syndrome; celiac disease; idiopathic pulmonary fibrosis	Phase I–IIa	CTSS mass accumulation; target redundancy;	(140, 151–153)
RWJ-445380	Small-molecule protease inhibitor reported as a CTSS-development candidate	Plaque psoriasis; rheumatoid arthritis	Phase II	Clinical outcomes and CTSS selectivity remain unverified; reasons for termination or non-progression were not reported.	(6, 151).
ASP1617	Orally active small-molecule inhibitor	SLE and lupus nephritis; clinical development evaluated in healthy subjects	Phase I	Lip10 accumulation; rebound CTSS activity; compensatory CTSS mass accumulation; dose-limiting adverse events.	(8, 9)
LY3000328	Orally active, noncovalent small-molecule inhibitor	Initially evaluated in healthy volunteers; subsequently used in cardiovascular and cancer models	Phase I	Systemic CTSS inhibition; rebound CTSS activity; prolonged CTSS mass accumulation; unestablished clinical efficacy; uncertain selectivity against related cathepsins.	(151, 154)
VBY-036	Small-molecule CTSS inhibitor	Neuropathic pain; Crohn’s disease	Phase I	Unreported pharmacodynamic and efficacy outcomes; unclear discontinuation reasons.	(151)
VBY-891	Small-molecule CTSS inhibitor	Neuropathic pain; psoriasis	Phase I	Efficacy and safety outcomes not reported; reasons for non-progression unclear.	(151)
CRA-028129	Small-molecule CTSS inhibitor;	Psoriasis	Phase I	Efficacy and safety outcomes not reported; reasons for non-progression unclear.	(155)
VBY-036	Small-molecule CTSS inhibitor;	Neuropathic pain	Phase I	Efficacy and safety outcomes not reported; reasons for non-progression unclear.	(155)
VBY-891	Small-molecule CTSS inhibitor;	Neuropathic pain; Psoriasis	Phase I	Efficacy and safety outcomes not reported; reasons for non-progression unclear.	(155)
VBY-825	Broad-spectrum, reversible covalent small-molecule cysteine cathepsin inhibitor	Neuropathic pain; bone cancer; autoimmune diseases; Alzheimer’s disease	Preclinical	Animal models; reduced NLRP3 activation, IL-1β production and gout inflammation; broad cathepsin inhibition; unclear CTSS-specific contribution; lack of clinical validation.	(156)
CA-074 Me	Cell-permeable, broad-spectrum small-molecule cysteine cathepsin inhibitor	MSU- and silica crystal-induced inflammation	Preclinical	Reduced crystal-induced IL-1β production and inflammatory-cell recruitment; broad cathepsin activity; unclear CTSS-specific contribution; lack of clinical validation.	(156)
CSI-75 (compound 5d)	Orally active, potent, selective and reversible small-molecule CTSS inhibitor	Rheumatoid arthritis and multiple sclerosis; T-cell-driven autoimmune diseases	Preclinical	Lip10 accumulation; suppressed antigen presentation and Th1/Th17 responses; lack of clinical validation.	(157)
MIV-247	Orally available small-molecule inhibitor	Neuropathic pain; autoimmune diseases; multiple sclerosis; rheumatoid arthritis	Preclinical	Animal models; cross-species CTSS potency; lack of clinical efficacy and safety data.	(151, 158)
Millipore-219393	Small-molecule CTSS inhibitor; also reported to stimulate PPARγ expression	Pulmonary arterial hypertension, including SLE-associated pulmonary hypertension	Preclinical	Animal models; Reduced CTSS activity and pulmonary vascular remodeling; PPARγ upregulation; lack of clinical validation; unestablished pharmacokinetics, safety and selectivity; uncertain contribution of PPARγ activation.	(94)
Anti-proCTSS vNAR	Shark-derived variable new antigen receptor single-domain antibodies; recombinant biologics and intracellularly expressed intrabodies	Experimental treatment of CTSS-driven cancer, particularly glioblastoma-cell invasion	Preclinical	CTSS activity and proCTSS maturation inhibition; reduced tumor-cell invasion; lack of *in vivo* validation; suboptimal intracellular delivery and binding affinity; unestablished pharmacokinetics, immunogenicity, and selectivity; inferior efficacy versus small-molecule inhibitors.	(148)

The available evidence does not deny the therapeutic potential of CTSS but indicates that indiscriminate systemic inhibition is unlikely to be universally effective. Successful clinical application will require the identification of CTSS-driven disease subsets and the alignment of treatment with the relevant cellular source, tissue compartment, substrate network, and disease stage (**Figure 4**).

**Figure 4:**
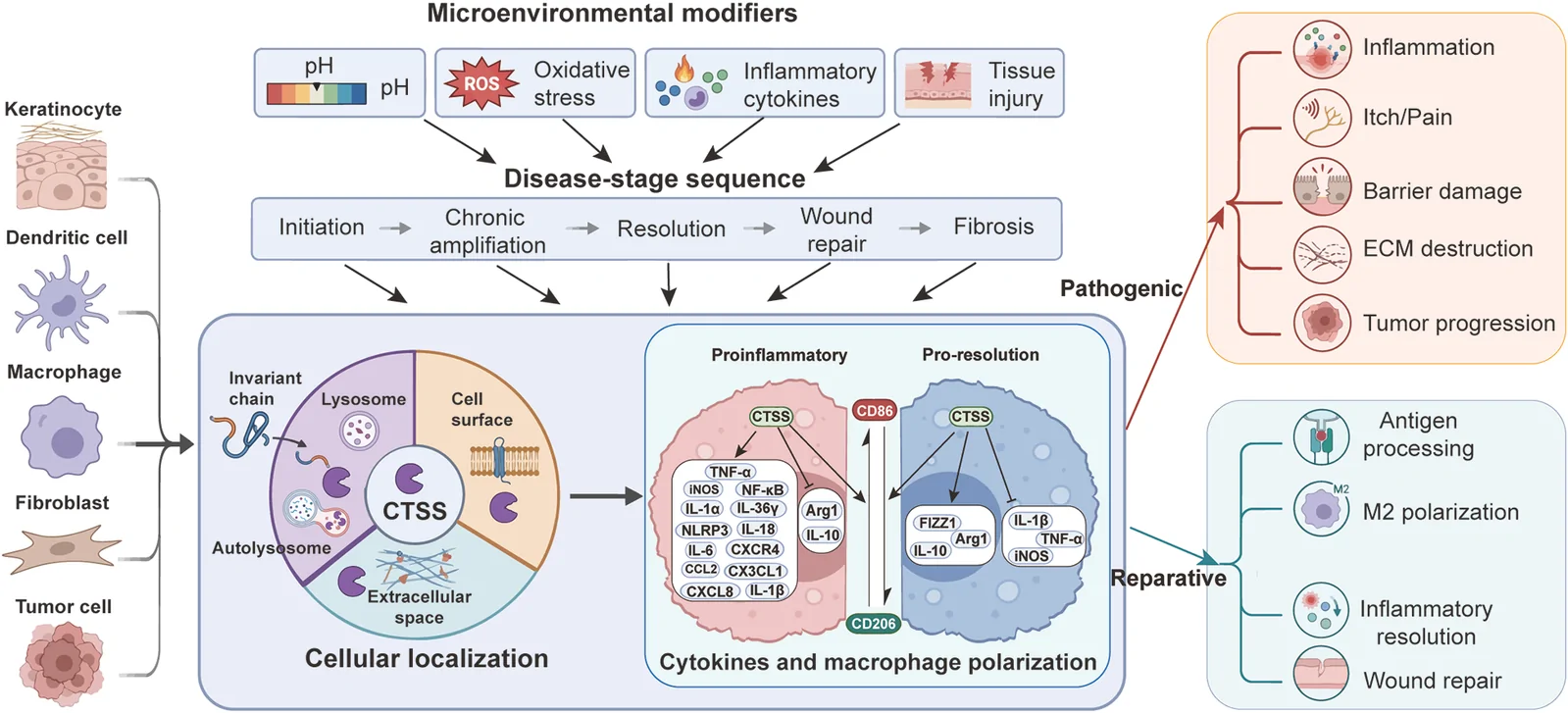
Context-dependent roles of CTSS in homeostasis and disease. The effects of CTSS depend on its cellular source, localization, substrates, microenvironment, and disease stage. CTSS may drive inflammation and tissue damage or promote immune homeostasis and repair, supporting disease-, compartment-, cell-, and stage-specific therapeutic strategies. The part of “Cytokines and macrophage polarization” was adapted from the referenced study (150) and it has been reproduced with permission from the original authors.

### 5.2 Possible factors contributing to limited efficacy of CTSS inhibitors

The limited efficacy or failure of CTSS inhibitors may result from several factors.

First, there is extensive substrate overlap and functional redundancy among cathepsins. CTSS, CTSK, and CTSL are papain-like cysteine cathepsins. Protease specificity profiling has shown that all three enzymes prefer hydrophobic residues at the P2 position, including Leu, Val, Ile, Phe, and Tyr (141). CTSS also exhibits certain specific features, such as relatively higher tolerance for P2 Lys, P1 Thr, and P1′ Ile (141). This substrate overlap helps explain why cathepsins readily compensate for one another and why highly selective CTSS inhibitor design remains challenging.

Second, CTSS and other cathepsins are not merely redundant parallel enzymes; they can also regulate each other through protease–protease interactions. The CTSS propeptide can inhibit CTSK, CTSL, and CTSV (10, 142, 143), and CTSS can degrade CTSK, thereby reducing CTSK-mediated type I collagen degradation (144). Because CTSS itself has relatively weak type I collagenolytic activity but retains elastinolytic activity, excessive CTSS may protect collagen-rich matrices by degrading CTSK while maintaining elastin degradation in elastin-rich tissues (144). This finding is particularly relevant to cutaneous ECM remodeling. CTSS may not simply promote global ECM degradation; rather, it may alter the balance between collagen and elastin degradation by regulating CTSK.

Third, cathepsin compensation networks may limit the efficacy of selective CTSS inhibition. A recent review emphasized that cathepsins function within complex proteolytic networks; when one cathepsin is genetically deleted or pharmacologically inhibited, other family members may compensate through increased expression, enhanced activity, or substrate substitution (145). The relationships among CTSS-CTSL, CTSS-CTSB, and CTSS-CTSK are especially relevant to CTSS biology (145). In dendritic cells and B cells, CTSS has a relatively nonredundant role in invariant chain processing; however, in macrophages and certain inflammatory settings, CTSL and CTSB may partially compensate for CTSS deficiency (145). IFN-γ may also shift the CTSL/CTSS activity balance, making antigen-presenting cells more dependent on CTSS for MHC-II antigen presentation (145). Therefore, in psoriasis, IL-36γ may still be activated by cathepsin G, CTSK, and neutrophil elastase even when CTSS is inhibited. In autoimmune diseases, CTSL, CTSB, or alternative antigen-processing pathways may partially compensate for CTSS-dependent MHC-II processing. Future drug development should therefore not rely solely on single-enzyme IC50 values, but should comprehensively evaluate the cathepsin network, cell type, pH environment, subcellular localization, and long-term compensatory responses.

Fourth, lysosomotropism is another key issue in CTSS inhibitor development. Lysosomotropic compounds can accumulate in acidic lysosomes and show strong apparent activity in cell-based assays. However, such compounds may also interact off-target with other cathepsins in different cell types or become ineffective when CTSS substrates are located in cytosolic or extracellular compartments (10). Inhibitor-induced retention of CTSS within lysosomes may also prevent redistribution of CTSS to extralysosomal sites, where CTSS regulates ECM remodeling and immune signaling. Because CTSS substrates are distributed across cellular compartments with distinct pH values, the protonation state of basic CTSS inhibitors should also be carefully evaluated (10). Therefore, future inhibitors should preferentially target specificity-determining regions, such as the S2 pocket, rather than the catalytic triad shared by cysteine cathepsins. Noncovalent inhibitors, reversible covalent inhibitors, fragment-based drug discovery, and nanocarrier-mediated delivery may improve selectivity and reduce off-target toxicity (10, 146).

### 5.3 Future CTSS-targeted strategies

Given these limitations, future CTSS-targeted therapy may need to shift from “systemic single-target inhibition” toward compartment-specific targeting, local delivery, selective blockade of downstream pathways, and combination therapy. Because skin lesions are directly accessible, local CTSS-targeted strategies have obvious advantages. Compared with systemic administration, topical or local delivery may reduce systemic immunosuppression while concentrating CTSS modulation within diseased skin. Potential formulations include topical gels or creams for inflammatory dermatoses, hydrogel dressings for acute wounds and chronic ulcers, microneedle-based delivery for dermal inflammation or scars, nanoparticle carriers for cell- or tissue-directed delivery, and enzyme-responsive delivery systems that preferentially release CTSS modulators in protease-rich inflammatory microenvironments (146). Nanocarrier-mediated delivery may be particularly useful when CTSS needs to be regulated in specific cellular compartments or immune-cell populations (146).

In addition to direct CTSS inhibition, targeting CTSS-modified downstream mediators may also be a feasible strategy. Because CTSS is multifunctional, global inhibition may block both pathogenic and protective functions. Therefore, selective intervention of downstream pathways modified by CTSS in specific diseases may be more appropriate. For example, CTSS inhibition could be combined with IL-36 blockade in psoriasis; CTSS inhibition could be combined with PAR2 antagonism in itch-dominant dermatoses; fractalkine/CX3CR1 signaling could be modulated in neuroimmune inflammation; and antimicrobial peptide-preserving strategies could be combined with CTSS modulation in barrier disorders (10). In wound repair, CTSS modulation should be approached more cautiously, because transient preservation or enhancement of CTSS activity may support macrophage-mediated resolution, angiogenesis, and matrix remodeling. In late-stage scarring or keloid formation, selective CTSS inhibition may help reduce excessive ECM remodeling. In melanoma, cell-specific targeting is required to suppress tumor invasion while avoiding impairment of antigen presentation and antitumor T-cell responses.

Recent evidence indicates that CTSS substrate specificity is dynamically regulated by environmental pH rather than determined solely by intrinsic catalytic activity. Around pH 7.2, CTSS undergoes a specificity switch driven by conformational rearrangement of Lys-64 into the S3 pocket, which restricts substrates carrying basic residues at the P3 position (147). This finding provides a mechanistic explanation for how CTSS may exert distinct functions across lysosomal, endosomal, and extracellular compartments. In inflammatory skin diseases, local pH changes may therefore shift CTSS from controlled immune signaling toward broader proteolytic tissue remodeling (147). From a therapeutic perspective, pH-dependent CTSS specificity supports the development of compartment- or microenvironment-selective inhibitors. Future drugs could selectively target extracellular or lysosomal CTSS conformations, particularly the pH-sensitive S3 pocket and Lys-64 gate, thereby improving efficacy while preserving physiological functions such as lysosomal antigen processing.

Emerging CTSS-targeting strategies also provide new directions for drug development. Recombinant CTSS propeptide can act as a subfamily-selective inhibitor of CTSL-like proteases, inhibiting CTSS, CTSL, CTSK, and CTSV while showing little inhibition of CTSB (142). This strategy suppresses CTSS-mediated elastin and gelatin degradation and reduces Matrigel invasion of multiple tumor cell lines; fusion to an Fc domain further improves extracellular stability and anti-invasive potency (142). This multi-target inhibition of CTSL-like proteases may help overcome protease compensation after selective CTSS inhibition. Another strategy is to use variable new antigen receptor (vNAR) domains to target pro-CTSS and block its maturation into the 25-kDa active enzyme, rather than directly inhibiting the active site of mature CTSS (148). Lead clones 3 and 5 bind pro-CTSS under acidic pH conditions, reduce CTSS maturation and enzymatic activity, and suppress U251 glioma cell invasion (148). This introduces the concept of blocking zymogen maturation as an alternative to conventional active-site inhibition. Nanocarrier-mediated delivery of CTSS inhibitors may also improve selectivity and therapeutic index, particularly in cancer immunotherapy. By targeting TAMs, DCs, or MDSCs, this approach may relieve myeloid immunosuppression while limiting systemic immune effects (146). In addition, computational drug design can facilitate CTSS inhibitor discovery. In the D3R Grand Challenge 3 CTSS subchallenge, multiscale weighted colored graph descriptors, element-specific persistent homology, random forest, and deep convolutional neural networks were integrated to predict CTSS ligand poses, affinity rankings, and binding free energies (149). Model performance improved substantially when crystallographic poses were available, indicating that docking accuracy remains a major bottleneck in CTSS virtual screening (149).

Finally, CTSS-targeted therapy requires reliable pharmacodynamic and patient-stratification biomarkers. CTSS mRNA or total protein levels do not necessarily reflect actual enzymatic activity. Future studies should measure active CTSS, substrate cleavage products, cystatin C levels, protease-inhibitor balance, ROS status, local pH, subcellular localization, and tissue-specific degradome signatures. In antigen-processing systems, Western blot detection of invariant chain p10 fragment accumulation has been used as a pharmacodynamic biomarker of CTSS inhibition (10). Emerging activity-based probes and degradomics platforms may enable direct mapping of CTSS–substrate–inhibitor interactions across different cellular compartments and disease stages. Future CTSS drug development should incorporate activity-based patient stratification, local delivery, combination therapy, and dynamic pharmacodynamic monitoring to improve efficacy while reducing systemic risks. Based on these mechanistic and clinical considerations, key context-aware strategies for the development of CTSS-targeted therapies are summarized in **Table 3**.

**Table 3:** Context-dependent strategies for developing CTSS-targeted therapies.

Strategic dimension	Key decision question	Recommended approach	Candidate biomarkers/readouts	Expected benefit	Main limitation or risk
Disease selection	Is CTSS a causal disease driver, a protective factor, or only a biomarker in the proposed indication?	Prioritize diseases with concordant human tissue, activity and functional evidence. Define the dominant CTSS-dependent pathway for each indication rather than extrapolating from CTSS expression alone.	Lesional CTSS activity; disease-relevant cleavage products; spatial colocalization with the implicated substrate/pathway; human-genetic or intervention evidence	Avoids treating disorders in which CTSS is non-causal or contributes to repair, immune surveillance or antitumor immunity.	Evidence remains associative or extrapolated in several dermatoses; CTSS may have opposing effects across diseases.
Patient stratification	Which patients have a CTSS-active molecular phenotype and are most likely to respond?	Use biomarker-enriched enrollment based on active CTSS and pathway-specific signatures; stratify by inflammatory, pruritic, fibrotic, wound-repair or tumor-immune phenotype.	Active CTSS rather than mRNA alone; CTSS mass; substrate fragments; cystatin C; local pH/ROS; IL-36, PAR2, CX3CL1 or degradome signatures	Enriches for CTSS-driven disease and improves interpretation of target engagement versus clinical response.	No validated predictive cutoffs; blood measurements may not reflect activity within skin lesions or specific compartments.
Pathogenic cellular source	Which cell population produces the disease-relevant CTSS?	Combine single-cell RNA sequencing, spatial transcriptomics, multiplex immunofluorescence and activity-based probes; functionally validate keratinocyte-, dendritic-cell-, macrophage-, neutrophil-, fibroblast-, endothelial- or tumor-cell-derived CTSS.	Cell type-specific CTSS transcript/protein/activity; proximity to substrates; cell-specific perturbation; immune and stromal-state markers	Enables cell-selective targeting and distinguishes pathogenic CTSS from CTSS supporting antigen presentation, repair or antitumor immunity.	Transcript abundance does not establish enzymatic activity; several cell types may contribute simultaneously or change over time.
Intracellular versus extracellular compartment	Is the relevant activity lysosomal, cell-surface/pericellular, or extracellular?	Map active CTSS and substrate cleavage by compartment. Develop extracellularly restricted, lysosome-directed or pH-aware inhibitors and consider targeting the pH-sensitive S3 pocket/Lys-64 gate.	Subcellular localization; active-site probe signal; invariant-chain p10 accumulation; extracellular cleavage products; compartmental pH	Preserves beneficial lysosomal antigen processing when extracellular proteolysis is pathogenic, or selectively targets intracellular activity when required.	Lysosomotropism, protonation and drug redistribution may cause off-target cathepsin inhibition or inadequate exposure at the relevant site.
Disease stage and therapeutic window	When is CTSS pathogenic, and when is it required for resolution or repair?	Define stage-specific profiles before intervention. Consider inhibition during chronic inflammatory amplification or late fibrotic/scarring phases, while avoiding indiscriminate suppression during early wound repair and inflammatory resolution.	Serial CTSS activity; inflammatory versus reparative macrophage markers; efferocytosis; angiogenesis; collagen/ECM turnover; wound-closure kinetics	Matches treatment timing to the changing function of CTSS and reduces interference with angiogenesis, M2 polarization and tissue repair.	Clinical stages overlap; the transition from pathogenic inflammation to reparative remodeling may be difficult to define.
Local or targeted delivery	Can effective exposure be confined to diseased skin, the relevant cell type and the relevant compartment?	Develop topical gels/creams, hydrogel dressings, microneedles, nanoparticles or enzyme-responsive carriers; optimize skin penetration, retention, release kinetics and cellular uptake for the intended target.	Skin and plasma pharmacokinetics; epidermal/dermal concentration; target-cell uptake; local CTSS inhibition; systemic exposure	Increases local therapeutic index and may reduce systemic immunosuppression and off-target effects.	The stratum corneum, rapid epidermal clearance, lesion heterogeneity and uncontrolled intracellular uptake may prevent adequate compartment-selective exposure.
Activity, mass and substrate monitoring	Does treatment inhibit active CTSS and the intended substrate pathway, rather than merely altering total protein abundance?	Measure enzymatic activity, mature CTSS, total CTSS mass and disease-relevant substrate cleavage longitudinally. Pair activity-based probes with degradomics and pharmacodynamic assays.	Active CTSS; mature/total CTSS; invariant-chain p10; disease-specific IL-36gamma, CX3CL1, ECM or other cleavage products; cystatin C; protease-inhibitor balance	Distinguishes pharmacological target engagement from compensatory protein accumulation and confirms pathway-level inhibition.	Assays are not standardized; substrate fragments may be generated by other proteases and may vary with pH and tissue compartment.
Combination therapy	Which parallel or downstream pathway sustains disease after CTSS inhibition?	Match combinations to disease biology: CTSS plus IL-36 blockade in psoriasis, PAR2 antagonism in itch-/dysesthesia-dominant dermatoses, CX3CL1/CX3CR1 modulation in neuroimmune inflammation, or barrier/antimicrobial-peptide-preserving treatment when appropriate.	Pathway-specific pharmacodynamics; cytokine and sensory scores; barrier measures; substrate cleavage; activity of alternative proteases	Addresses pathway redundancy while allowing lower or more localized CTSS inhibition.	Added toxicity and trial complexity; combinations must not simultaneously suppress protective immune, antimicrobial or reparative functions.
Rebound and compensatory-network surveillance	Does sustained or withdrawn treatment increase CTSS mass/activity or redirect substrates to other proteases?	Use serial on-treatment and post-treatment sampling. Profile CTSS, CTSL, CTSB, CTSK, cathepsin G, neutrophil elastase, kallikreins, MMPs and serine proteases, together with shared substrate products.	CTSS activity and mass; Lip10/p10 accumulation; alternative-protease activity; IL-36gamma processing; ECM degradome; post-withdrawal activity trajectory	Detects loss of efficacy, rebound and substrate substitution early and informs dosing schedules or rational multi-pathway therapy.	Protease networks are cell-, pH- and time-dependent; broad inhibition may increase off-target toxicity and disturb physiological proteolysis.
Long-term immune, infection and repair safety	Does chronic CTSS modulation impair antigen presentation, host defense, immune surveillance, wound healing or antitumor immunity?	Build longitudinal safety assessments into trials, including infection surveillance, vaccine/immune-response measures, wound-healing evaluation and tumor-immune monitoring; use the lowest effective local exposure and predefined interruption criteria.	Serious and opportunistic infections; immunoglobulin/immune-response measures; barrier integrity; antimicrobial peptides; wound closure and scar quality; tumor immune infiltration	Protects against delayed adverse effects that short target-engagement studies may miss.	Some beneficial CTSS functions are compartment- and stage-specific; uncommon immune or repair complications require longer and larger studies.

## 6 Future perspectives

Several key questions remain unresolved. The cellular sources of CTSS in different skin diseases need to be defined. Single-cell RNA sequencing, spatial transcriptomics, multiplex immunofluorescence, and activity-based probes should be used to determine whether CTSS derives mainly from keratinocytes, dendritic cells, macrophages, neutrophils, endothelial cells, fibroblasts, sensory nerves, or tumor cells. CTSS expression should be distinguished from CTSS activity. Future studies should measure mature active CTSS, enzymatic activity, substrate cleavage products, inhibitor balance, pH, ROS, and extracellular localization. CTSS should be studied within protease networks. Other cathepsins, kallikreins, neutrophil elastase, matrix metalloproteinases, and serine proteases may compensate for or cooperate with CTSS. This may explain why CTSS inhibitor monotherapy has shown limited efficacy in psoriasis and other diseases. The CTSS-PAR2 and CTSS-fractalkine axes should be explored in other chronic pruritic dermatoses such as chronic prurigo, uremic pruritus, cholestatic pruritus, neuropathic itch, or post-burn itch remains unclear. The role of CTSS in macrophage efferocytosis and inflammatory resolution deserves further investigation. CTSS is required for autophagy and M2 polarization, and macrophage-mediated clearance of apoptotic cells is essential for wound repair. Whether CTSS regulates MerTK expression, MerTK phosphorylation, lysosome-associated phagocytosis, apoptotic neutrophil clearance, and IL-10/TGF-β production in skin wounds is an important future direction. CTSS-mediated immune tolerance mechanisms warrant further investigation in other autoimmune dermatoses, such as alopecia areata, autoimmune blistering diseases, and other T-cell-mediated dermatoses.

Future drug-development efforts should determine how extracellular CTSS can be selectively targeted without impairing lysosomal CTSS-dependent antigen presentation and immune surveillance. Topical formulations, extracellularly restricted inhibitors, pH-aware compounds, and nanocarrier-based delivery systems may help achieve compartment-selective modulation. In melanoma, CTSS pathway activity should be evaluated as a potential predictor of tumor immune infiltration and immunotherapy response, given its dual association with ECM invasion and antigen presentation. Finally, artificial intelligence-based prediction of CTSS substrate spectra, cleavage sites, protease interaction networks, and disease-specific degradomes may accelerate identification of CTSS functions and facilitate translational development.

## 7 Conclusion

CTSS is a multifunctional cysteine protease that integrates lysosomal proteostasis, antigen presentation, immune tolerance, inflammatory signaling, neuroimmune activation, antimicrobial defense, ECM remodeling, angiogenesis, autophagy, macrophage polarization, fibrosis, wound healing, and tumor immunity. In skin diseases, CTSS acts as a protease hub linking barrier dysfunction, neuroimmune inflammation, immune tolerance, antimicrobial defense, and tissue remodeling. It contributes to psoriasis through IL-36γ activation and keratinocyte-associated antigen presentation; to AD and seborrheic dermatitis through PAR2-mediated itch and inflammation; to autoimmune diseases through MHC-II-dependent CD4^+^ T cell and B cell activation and potential tolerance disruption; to wound healing and scarring through ECM remodeling, angiogenesis, macrophage function, and collagen regulation; and to melanoma through both tumor-promoting and antitumor immune mechanisms.

## Glossary

3′UTR: 3′ untranslated region
AD: atopic dermatitis
ADAM: a disintegrin and metalloproteinase
AITC: allyl isothiocyanate
Akt: protein kinase B
ATF3: activating transcription factor 3
ATP: adenosine triphosphate
BAF60a: BRG1/BRM-associated factor 60a
BCG: Bacillus Calmette–Guérin
BDNF: brain-derived neurotrophic factor
BMI: body mass index
Blimp-1: B lymphocyte-induced maturation protein 1
BRCA1: breast cancer susceptibility gene 1
BRG1: Brahma-related gene 1
cAMP: cyclic adenosine monophosphate
CTS: cathepsin
CCL: C-C motif chemokine ligand
CCR: C-C motif chemokine receptor
CD: cluster of differentiation
CIBERSORT: Cell-type Identification By Estimating Relative Subsets Of RNA Transcripts
CLIP: class II-associated invariant chain peptide
CM: cutaneous melanoma
CX3CL1: C-X3-C motif chemokine ligand 1
CX3CR1: C-X3-C motif chemokine receptor 1
CXCL: C-X-C motif chemokine ligand
CXCR4: C-X-C motif chemokine receptor 4
D3R: Drug Design Data Resource
DCs: dendritic cells
DNA: deoxyribonucleic acid
DRG: dorsal root ganglion
ECM: extracellular matrix
EphB2: Eph receptor B2
ERK: extracellular signal-regulated kinase
Gαs: G protein alpha subunit
GSDMD: gasdermin D
GsdmC: gasdermin C
HLA-DM: human leukocyte antigen DM
HuR: human antigen R
IC50: half-maximal inhibitory concentration
IFN: interferon
IL: interleukin
IRF: interferon regulatory factor
JAK: Janus kinase
LAMP3: lysosome-associated membrane glycoprotein 3
LAP: LC3-associated phagocytosis
LC3: microtubule-associated protein 1 light chain 3
LL-37: cathelicidin antimicrobial peptide LL-37
MAPK: mitogen-activated protein kinase
MCP-1: monocyte chemoattractant protein-1
MDSCs: myeloid-derived suppressor cells
MerTK: Mer receptor tyrosine kinase
MHC: major histocompatibility complex
miR-31: microRNA-31
MMP: matrix metalloproteinase
MRGPR: Mas-related G protein-coupled receptor
mRNA: messenger RNA
mTORC1: mechanistic target of rapamycin complex 1
NADPH: reduced nicotinamide adenine dinucleotide phosphate
NF-κB: nuclear factor kappa B
P2X4: purinergic receptor P2X4
P2X7: purinergic receptor P2X7
PAR2: protease-activated receptor 2
PASI75: 75% improvement in Psoriasis Area and Severity Index
PI3K: phosphoinositide 3-kinase
PKA: protein kinase A
PKC: protein kinase C
PP2A: protein phosphatase 2A
PPARγ: peroxisome proliferator-activated receptor gamma
proCTSS/pro-CTSS: pro-cathepsin S
PU.1: purine-rich box 1 transcription factor
Rab7: Ras-related protein Rab-7a
Raf: rapidly accelerated fibrosarcoma kinase
Ras: rat sarcoma viral oncogene homolog
RNA: ribonucleic acid
RNA-seq: RNA sequencing
ROS: reactive oxygen species
SFRP2: secreted frizzled-related protein 2
SIRT1: sirtuin 1
SLE: systemic lupus erythematosus
SLPI: secretory leukoprotease inhibitor
Smad: small mothers against decapentaplegic
STAT: signal transducer and activator of transcription
TAMs: tumor-associated macrophages
TAP: transporter associated with antigen processing
TFEB: transcription factor EB
TGF-β: transforming growth factor beta
Th1: T helper 1
TLR: Toll-like receptor
TNF: tumor necrosis factor
TRPV: transient receptor potential vanilloid
UTR: untranslated region
vNAR: variable new antigen receptor
α2δ: alpha-2-delta subunit of voltage-gated calcium channels

## References

[1] Hooper NM . Proteases: a primer. Essays Biochem. (2002) 38:1–8. doi: 10.1042/bse0380001 12463157

[2] Smyth P Sasiwachirangkul J Williams R Scott CJ . Cathepsin S (CTSS) activity in health and disease - a treasure trove of untapped clinical potential. Mol Aspects Med. (2022) 88:101106. doi: 10.1016/j.mam.2022.101106 35868042

[3] Patel S Homaei A El-Seedi HR Akhtar N . Cathepsins: proteases that are vital for survival but can also be fatal. BioMed Pharmacother. (2018) 105:526–32. doi: 10.1016/j.biopha.2018.05.148 29885636 PMC7172164

[4] Vidak E Javorsek U Vizovisek M Turk B . Cysteine cathepsins and their extracellular roles: shaping the microenvironment. Cells. (2019) 8:264. doi: 10.3390/cells8030264 30897858 PMC6468544

[5] Gao H Zhang Z Deng J Song Y . Cathepsin S: molecular mechanisms in inflammatory and immunological processes. Front Immunol. (2025) 16:1600206. doi: 10.3389/fimmu.2025.1600206 40692794 PMC12277131

[6] Yoo Y Choi E Kim Y Cha Y Um E Kim Y . Therapeutic potential of targeting cathepsin S in pulmonary fibrosis. BioMed Pharmacother. (2022) 145:112245. doi: 10.1016/j.biopha.2021.112245 34772578

[7] David A Chazeirat T Saidi A Lalmanach G Lecaille F . The interplay of glycosaminoglycans and cysteine cathepsins in mucopolysaccharidosis. Biomedicines. (2023) 11:810. doi: 10.3390/biomedicines11030810 36979788 PMC10045161

[8] Kawato Y Fukahori H Nakamura K Kanno A Kubo K Hiramitsu M . Potential benefit of the cathepsin S inhibitor, ASP1617, as a treatment for systemic lupus erythematosus. Eur J Pharmacol. (2022) 919:174826. doi: 10.1016/j.ejphar.2022.174826 35157914

[9] Kojima T Chen W Smulders R Stanhope S Kowalski D Heo N . First-in-human study to evaluate the safety, tolerability, pharmacokinetics, and pharmacodynamics of ASP1617, a cathepsin-S inhibitor, in healthy adult subjects. Clin Transl Sci. (2026) 19:e70460. doi: 10.1111/cts.70460 41577633 PMC12830235

[10] Geetha D Skaria T . Cathepsin S: a key drug target and signalling hub in immune system diseases. Int Immunopharmacol. (2025) 155:114622. doi: 10.1016/j.intimp.2025.114622 40220622

[11] Veilleux A Black WC Gauthier JY Mellon C Percival MD Tawa P . Probing cathepsin S activity in whole blood by the activity-based probe BIL-DMK: cellular distribution in human leukocyte populations and evidence of diurnal modulation. Anal Biochem. (2011) 411:43–9. doi: 10.1016/j.ab.2010.11.022 21094118

[12] Vasiljeva O Dolinar M Pungercar JR Turk V Turk B . Recombinant human procathepsin S is capable of autocatalytic processing at neutral pH in the presence of glycosaminoglycans. FEBS Lett. (2005) 579:1285–90. doi: 10.1016/j.febslet.2004.12.093 15710427

[13] Brix K Dunkhorst A Mayer K Jordans S . Cysteine cathepsins: cellular roadmap to different functions. Biochimie. (2008) 90:194–207. doi: 10.1016/j.biochi.2007.07.024 17825974

[14] Rodriguez A Webster P Ortego J Andrews NW . Lysosomes behave as Ca2+-regulated exocytic vesicles in fibroblasts and epithelial cells. J Cell Biol. (1997) 137:93–104. doi: 10.1083/jcb.137.1.93 9105039 PMC2139854

[15] Yan D Wang HW Bowman RL Joyce JA . STAT3 and STAT6 signaling pathways synergize to promote cathepsin secretion from macrophages via IRE1alpha activation. Cell Rep. (2016) 16:2914–27. doi: 10.1016/j.celrep.2016.08.035 27626662 PMC5559199

[16] Kirschke H Wiederanders B Bromme D Rinne A . Cathepsin S from bovine spleen. Purification, distribution, intracellular localization and action on proteins. Biochem J. (1989) 264:467–73. doi: 10.1042/bj2640467 2690828 PMC1133603

[17] Novinec M Lenarcic B Turk B . Cysteine cathepsin activity regulation by glycosaminoglycans. BioMed Res Int. (2014) 2014:309718. doi: 10.1155/2014/309718 25587532 PMC4283429

[18] Wang H Meng X Piao L Inoue A Xu W Yu C . Cathepsin S deficiency mitigated chronic stress-related neointimal hyperplasia in mice. J Am Heart Assoc. (2019) 8:e011994. doi: 10.1161/JAHA.119.011994 31296090 PMC6662117

[19] Xu S Piao L Wan Y Huang Z Meng X Inoue A . CTSS modulates stress-related carotid artery thrombosis in a mouse FeCl(3) model. Arterioscler Thromb Vasc Biol. (2023) 43:e238–53. doi: 10.1161/ATVBAHA.122.318455 37128920

[20] Sayed S Faruq O Preya UH Kim JT . Cathepsin S knockdown suppresses endothelial inflammation, angiogenesis, and complement protein activity under hyperglycemic conditions *in vitro* by inhibiting NF-kappaB signaling. Int J Mol Sci. (2023) 24:5428. doi: 10.3390/ijms24065428 36982499 PMC10049538

[21] Ni H Xu S Chen H Dai Q . Nicotine modulates CTSS (cathepsin S) synthesis and secretion through regulating the autophagy-lysosomal machinery in atherosclerosis. Arterioscler Thromb Vasc Biol. (2020) 40:2054–69. doi: 10.1161/ATVBAHA.120.314053 32640907

[22] Doherty DF Nath S Poon J Foronjy RF Ohlmeyer M Dabo AJ . Protein phosphatase 2A reduces cigarette smoke-induced cathepsin S and loss of lung function. Am J Respir Crit Care Med. (2019) 200:51–62. doi: 10.1164/rccm.201808-1518OC 30641028 PMC6603057

[23] Wu H Du Q Dai Q Ge J Cheng X . Cysteine protease cathepsins in atherosclerotic cardiovascular diseases. J Atheroscler Thromb. (2018) 25:111–23. doi: 10.5551/jat.RV17016 28978867 PMC5827079

[24] Magister S Obermajer N Mirkovic B Svajger U Renko M Softic A . Regulation of cathepsins S and L by cystatin F during maturation of dendritic cells. Eur J Cell Biol. (2012) 91:391–401. doi: 10.1016/j.ejcb.2012.01.001 22365146

[25] Haves-Zburof D Paperna T Gour-Lavie A Mandel I Glass-Marmor L Miller A . Cathepsins and their endogenous inhibitors cystatins: expression and modulation in multiple sclerosis. J Cell Mol Med. (2011) 15:2421–9. doi: 10.1111/j.1582-4934.2010.01229.x 21143385 PMC3822953

[26] Steimle A Gronbach K Beifuss B Schafer A Harmening R Bender A . Symbiotic gut commensal bacteria act as host cathepsin S activity regulators. J Autoimmun. (2016) 75:82–95. doi: 10.1016/j.jaut.2016.07.009 27484364

[27] Xu Y Ding Y Li X Wu X . Cystatin C is a disease-associated protein subject to multiple regulation. Immunol Cell Biol. (2015) 93:442–51. doi: 10.1038/icb.2014.121 25643616 PMC7165929

[28] Wartenberg M Andrault PM Saidi A Bigot P Nadal-Desbarats L Lecaille F . Oxidation of cathepsin S by major chemicals of cigarette smoke. Free Radic Biol Med. (2020) 150:53–65. doi: 10.1016/j.freeradbiomed.2020.02.013 32084513

[29] Storm van's Gravesande K Layne MD Ye Q Le L Baron RM Perrella MA . IFN regulatory factor-1 regulates IFN-gamma-dependent cathepsin S expression. J Immunol. (2002) 168:4488–94. doi: 10.4049/jimmunol.168.9.4488 11970993

[30] Yin J Shi D Sun Y Zhu P Zhao Y Xu X . IRF7 and CTSS are pivotal for cutaneous wound healing and may serve as therapeutic targets. Signal Transduct Target Ther. (2023) 8:322. doi: 10.1038/s41392-023-01517-1 37644004 PMC10465541

[31] Chen DJ Tang L Chen B Fu SY Fan MJ Chen GJ . IRF8-cathepsin S/PAR2 axis-mediated spinal microglia-neuron crosstalk is responsible for the exacerbation of postsurgical pain induced by preoperative stress. Int Immunopharmacol. (2025) 161:115034. doi: 10.1016/j.intimp.2025.115034 40482455

[32] Wang Y Baron RM Zhu G Joo M Christman JW Silverman ES . PU.1 regulates cathepsin S expression in professional APCs. J Immunol. (2006) 176:275–83. doi: 10.4049/jimmunol.176.1.275 16365419

[33] Zhang K Wang S Wang Z Jiang Y Huang M Liu N . Critical roles of PU.1/cathepsin S activation in regulating inflammatory responses of macrophages in periodontitis. J Periodontal Res. (2023) 58:939–47. doi: 10.1111/jre.13153 37334752

[34] Kim SJ Schatzle S Ahmed SS Haap W Jang SH Gregersen PK . Increased cathepsin S in Prdm1(-/-) dendritic cells alters the T(FH) cell repertoire and contributes to lupus. Nat Immunol. (2017) 18:1016–24. doi: 10.1038/ni.3793 28692065 PMC5568473

[35] Ji C Tang M Harrison J Paciorkowski A Johnson GVW . Nuclear transglutaminase 2 directly regulates expression of cathepsin S in rat cortical neurons. Eur J Neurosci. (2018) 48:3043–51. doi: 10.1111/ejn.14159 30239049

[36] Kitamura H Kamon H Sawa S Park SJ Katunuma N Ishihara K . IL-6-STAT3 controls intracellular MHC class II alphabeta dimer level through cathepsin S activity in dendritic cells. Immunity. (2005) 23:491–502. doi: 10.1016/j.immuni.2005.09.010 16286017

[37] Chan LL Cheung BK Li JC Lau AS . A role for STAT3 and cathepsin S in IL-10 down-regulation of IFN-gamma-induced MHC class II molecule on primary human blood macrophages. J Leukoc Biol. (2010) 88:303–11. doi: 10.1189/jlb.1009659 20356901

[38] Gautam J Bae YK Kim JA . Up-regulation of cathepsin S expression by HSP90 and 5-HT(7) receptor-dependent serotonin signaling correlates with triple negativity of human breast cancer. Breast Cancer Res Treat. (2017) 161:29–40. doi: 10.1007/s10549-016-4027-1 27796714

[39] Chang CJ Hsu HC Ho WJ Chang GJ Pang JS Chen WJ . Cathepsin S promotes the development of pulmonary arterial hypertension. Am J Physiol Lung Cell Mol Physiol. (2019) 317:L1–L13. doi: 10.1152/ajplung.00530.2018 31017016

[40] Zhang X Zhou Y Yu X Huang Q Fang W Li J . Differential roles of cysteinyl cathepsins in TGF-beta signaling and tissue fibrosis. iScience. (2019) 19:607–22. doi: 10.1016/j.isci.2019.08.014 31446224 PMC6715892

[41] Stellos K Gatsiou A Stamatelopoulos K Perisic Matic L John D Lunella FF . Adenosine-to-inosine RNA editing controls cathepsin S expression in atherosclerosis by enabling HuR-mediated post-transcriptional regulation. Nat Med. (2016) 22:1140–50. doi: 10.1038/nm.4172 27595325

[42] Weldon S McNally P McAuley DF Oglesby IK Wohlford-Lenane CL Bartlett JA . miR-31 dysregulation in cystic fibrosis airways contributes to increased pulmonary cathepsin S production. Am J Respir Crit Care Med. (2014) 190:165–74. doi: 10.1164/rccm.201311-1986OC 24940638 PMC4226050

[43] Clark AK Wodarski R Guida F Sasso O Malcangio M . Cathepsin S release from primary cultured microglia is regulated by the P2X7 receptor. Glia. (2010) 58:1710–26. doi: 10.1002/glia.21042 20629190

[44] Cao L Cao X Zhou Y Nagpure BV Wu ZY Hu LF . Hydrogen sulfide inhibits ATP-induced neuroinflammation and Abeta(1-42) synthesis by suppressing the activation of STAT3 and cathepsin S. Brain Behav Immun. (2018) 73:603–14. doi: 10.1016/j.bbi.2018.07.005 29981830

[45] Zhou YB Zhou H Li L Kang Y Cao X Wu ZY . Hydrogen sulfide prevents elastin loss and attenuates calcification induced by high glucose in smooth muscle cells through suppression of Stat3/cathepsin S signaling pathway. Int J Mol Sci. (2019) 20:4202. doi: 10.3390/ijms20174202 31461977 PMC6747320

[46] Huang J Chang Z Liu L Lai C Deng X Jiang B . Maraviroc prevents optic nerve injury-induced retinal ganglion cell apoptosis by modulating the CCL5/CCR5/CTSS axis. Invest Ophthalmol Vis Sci. (2025) 66:77. doi: 10.1167/iovs.66.6.77 40557874 PMC12205568

[47] Nakagawa TY Brissette WH Lira PD Griffiths RJ Petrushova N Stock J . Impaired invariant chain degradation and antigen presentation and diminished collagen-induced arthritis in cathepsin S null mice. Immunity. (1999) 10:207–17. doi: 10.1016/s1074-7613(00)80021-7 10072073

[48] Roche PA Furuta K . The ins and outs of MHC class II-mediated antigen processing and presentation. Nat Rev Immunol. (2015) 15:203–16. doi: 10.1038/nri3818 25720354 PMC6314495

[49] Jurewicz MM Stern LJ . Class II MHC antigen processing in immune tolerance and inflammation. Immunogenetics. (2019) 71:171–87. doi: 10.1007/s00251-018-1095-x 30421030 PMC6377339

[50] Stoeckle C Quecke P Ruckrich T Burster T Reich M Weber E . Cathepsin S dominates autoantigen processing in human thymic dendritic cells. J Autoimmun. (2012) 38:332–43. doi: 10.1016/j.jaut.2012.02.003 22424724

[51] Shen L Sigal LJ Boes M Rock KL . Important role of cathepsin S in generating peptides for TAP-independent MHC class I crosspresentation *in vivo*. Immunity. (2004) 21:155–65. doi: 10.1016/j.immuni.2004.07.004 15308097

[52] Colbert JD Cruz FM Rock KL . Cross-presentation of exogenous antigens on MHC I molecules. Curr Opin Immunol. (2020) 64:1–8. doi: 10.1016/j.coi.2019.12.005 31927332 PMC7343603

[53] Manoury B . TLR9 regulation by proteolysis: a friend or a foe. Eur J Immunol. (2011) 41:2142–4. doi: 10.1002/eji.201141858 21792872

[54] Orlowski GM Colbert JD Sharma S Bogyo M Robertson SA Rock KL . Multiple cathepsins promote Pro-IL-1beta synthesis and NLRP3-mediated IL-1beta activation. J Immunol. (2015) 195:1685–97. doi: 10.4049/jimmunol.1500509 26195813 PMC4530060

[55] de Mingo A de Gregorio E Moles A Tarrats N Tutusaus A Colell A . Cysteine cathepsins control hepatic NF-kappaB-dependent inflammation via sirtuin-1 regulation. Cell Death Dis. (2016) 7:e2464. doi: 10.1038/cddis.2016.368 27831566 PMC5260902

[56] Flynn CM Garbers Y Dusterhoft S Wichert R Lokau J Lehmann CHK . Cathepsin S provokes interleukin-6 (IL-6) trans-signaling through cleavage of the IL-6 receptor *in vitro*. Sci Rep. (2020) 10:21612. doi: 10.1038/s41598-020-77884-4 33303781 PMC7730449

[57] Ainscough JS Macleod T McGonagle D Brakefield R Baron JM Alase A . Cathepsin S is the major activator of the psoriasis-associated proinflammatory cytokine IL-36gamma. Proc Natl Acad Sci USA. (2017) 114:E2748–57. doi: 10.1073/pnas.1620954114 28289191 PMC5380102

[58] Mercurio L Failla CM Capriotti L Scarponi C Facchiano F Morelli M . Interleukin (IL)-17/IL-36 axis participates to the crosstalk between endothelial cells and keratinocytes during inflammatory skin responses. PloS One. (2020) 15:e0222969. doi: 10.1371/journal.pone.0222969 32352958 PMC7192413

[59] Yuan ZC Xu WD Liu XY Liu XY Huang AF Su LC . Biology of IL-36 signaling and its role in systemic inflammatory diseases. Front Immunol. (2019) 10:2532. doi: 10.3389/fimmu.2019.02532 31736959 PMC6839525

[60] Li N Yamasaki K Saito R Fukushi-Takahashi S Shimada-Omori R Asano M . Alarmin function of cathelicidin antimicrobial peptide LL37 through IL-36gamma induction in human epidermal keratinocytes. J Immunol. (2014) 193:5140–8. doi: 10.4049/jimmunol.1302574 25305315

[61] Chen F Tian XF Yang T Dai YJ Chen DY Chen HB . Lysosomal cathepsin S escape facilitates near infrared light-triggered pyroptosis via an antibody-indocyanine green conjugate. Adv Sci (Weinh). (2025) 12:e04851. doi: 10.1002/advs.202504851 40539828 PMC12442674

[62] Pandey SP Yang D Hedden L Laughlin CR Wang W Soto AS . Gasdermin C cleavage by cathepsin S modulates Rab7 vesicles in intestinal epithelial cells to amplify anti-helminth immunity. Immunity. (2025) 58:2439–55:e8. doi: 10.1016/j.immuni.2025.06.018 40701157 PMC12380176

[63] Chung K Pitcher T Grant AD Hewitt E Lindstrom E Malcangio M . Cathepsin S acts via protease-activated receptor 2 to activate sensory neurons and induce itch-like behaviour. Neurobiol Pain. (2019) 6:100032. doi: 10.1016/j.ynpai.2019.100032 31223140 PMC6565756

[64] Zhao P Lieu T Barlow N Metcalf M Veldhuis NA Jensen DD . Cathepsin S causes inflammatory pain via biased agonism of PAR2 and TRPV4. J Biol Chem. (2014) 289:27215–34. doi: 10.1074/jbc.M114.599712 25118282 PMC4175355

[65] Fonovic UP Jevnikar Z Kos J . Cathepsin S generates soluble CX3CL1 (fractalkine) in vascular smooth muscle cells. Biol Chem. (2013) 394:1349–52. doi: 10.1515/hsz-2013-0189 23893684

[66] Yang M Liu J Shao J Qin Y Ji Q Zhang X . Cathepsin S-mediated autophagic flux in tumor-associated macrophages accelerate tumor development by promoting M2 polarization. Mol Cancer. (2014) 13:43. doi: 10.1186/1476-4598-13-43 24580730 PMC4015740

[67] Levin AD Koelink PJ Bloemendaal FM Vos AC D'Haens GR van den Brink GR . Autophagy contributes to the induction of anti-TNF induced macrophages. J Crohns Colitis. (2016) 10:323–9. doi: 10.1093/ecco-jcc/jjv174 26417049 PMC4957464

[68] Fei M Zhang L Wang H Zhu Y Niu W Tang T . Inhibition of cathepsin S induces mitochondrial apoptosis in glioblastoma cell lines through mitochondrial stress and autophagosome accumulation. Front Oncol. (2020) 10:516746. doi: 10.3389/fonc.2020.516746 33425712 PMC7787074

[69] De Leo MG Staiano L Vicinanza M Luciani A Carissimo A Mutarelli M . Autophagosome-lysosome fusion triggers a lysosomal response mediated by TLR9 and controlled by OCRL. Nat Cell Biol. (2016) 18:839–50. doi: 10.1038/ncb3386 27398910 PMC5040511

[70] Wang H Jiang H Cheng XW . Cathepsin S are involved in human carotid atherosclerotic disease progression, mainly by mediating phagosomes: bioinformatics and *in vivo* and vitro experiments. PeerJ. (2022) 10:e12846. doi: 10.7717/peerj.12846 35186462 PMC8833225

[71] Wan Y Piao L Xu S Inoue A Meng X Lei Y . Cathepsin S deficiency improves muscle mass loss and dysfunction via the modulation of protein metabolism in mice under pathological stress conditions. FASEB J. (2023) 37:e23086. doi: 10.1096/fj.202300395RRR 37428652

[72] Boada-Romero E Martinez J Heckmann BL Green DR . The clearance of dead cells by efferocytosis. Nat Rev Mol Cell Biol. (2020) 21:398–414. doi: 10.1038/s41580-020-0232-1 32251387 PMC7392086

[73] Hayashi K Taura M Iwasaki A . The interaction between IKKalpha and LC3 promotes type I interferon production through the TLR9-containing LAPosome. Sci Signal. (2018) 11:eaan4144. doi: 10.1126/scisignal.aan4144 29717061 PMC6462218

[74] Vizovisek M Fonovic M Turk B . Cysteine cathepsins in extracellular matrix remodeling: Extracellular matrix degradation and beyond. Matrix Biol. (2019) 75-76:141–59. doi: 10.1016/j.matbio.2018.01.024 29409929

[75] Sobotic B Vizovisek M Vidmar R Van Damme P Gocheva V Joyce JA . Proteomic identification of cysteine cathepsin substrates shed from the surface of cancer cells. Mol Cell Proteomics. (2015) 14:2213–28. doi: 10.1074/mcp.M114.044628 26081835 PMC4528248

[76] Veillard F Saidi A Burden RE Scott CJ Gillet L Lecaille F . Cysteine cathepsins S and L modulate anti-angiogenic activities of human endostatin. J Biol Chem. (2011) 286:37158–67. doi: 10.1074/jbc.M111.284869 21896479 PMC3199463

[77] Schwarz G Boehncke WH Braun M Schroter CJ Burster T Flad T . Cathepsin S activity is detectable in human keratinocytes and is selectively upregulated upon stimulation with interferon-gamma. J Invest Dermatol. (2002) 119:44–9. doi: 10.1046/j.1523-1747.2002.01800.x 12164923

[78] Medovic MV Jakovljevic VL Zivkovic VI Jeremic NS Jeremic JN Bolevich SB . Psoriasis between autoimmunity and oxidative stress: Changes induced by different therapeutic approaches. Oxid Med Cell Longev. (2022) 2022:2249834. doi: 10.1155/2022/2249834 35313642 PMC8934232

[79] Ma F Plazyo O Billi AC Tsoi LC Xing X Wasikowski R . Single cell and spatial sequencing define processes by which keratinocytes and fibroblasts amplify inflammatory responses in psoriasis. Nat Commun. (2023) 14:3455. doi: 10.1038/s41467-023-39020-4 37308489 PMC10261041

[80] Taggart CC Greene CM Smith SG Levine RL McCray PB Jr. O'Neill S . Inactivation of human beta-defensins 2 and 3 by elastolytic cathepsins. J Immunol. (2003) 171:931–7. doi: 10.4049/jimmunol.171.2.931 12847264

[81] Andrault PM Samsonov SA Weber G Coquet L Nazmi K Bolscher JG . Antimicrobial peptide LL-37 is both a substrate of cathepsins S and K and a selective inhibitor of cathepsin L. Biochemistry. (2015) 54:2785–98. doi: 10.1021/acs.biochem.5b00231 25884905

[82] Kim N Bae KB Kim MO Yu DH Kim HJ Yuh HS . Overexpression of cathepsin S induces chronic atopic dermatitis in mice. J Invest Dermatol. (2012) 132:1169–76. doi: 10.1038/jid.2011.404 22170489

[83] Klinngam W Fu R Janga SR Edman MC Hamm-Alvarez SF . Cathepsin S alters the expression of pro-inflammatory cytokines and MMP-9, partially through protease-activated receptor-2, in human corneal epithelial cells. Int J Mol Sci. (2018) 19:3530. doi: 10.3390/ijms19113530 30423938 PMC6274678

[84] Reddy VB Sun S Azimi E Elmariah SB Dong X Lerner EA . Redefining the concept of protease-activated receptors: cathepsin S evokes itch via activation of Mrgprs. Nat Commun. (2015) 6:7864. doi: 10.1038/ncomms8864 26216096 PMC4520244

[85] Singto T Sergeeva A Filor V Vidak J Kleuser B Belik V . Immune cells in dorsal root ganglia are associated with pruritus in a mouse model of allergic contact dermatitis and co-culture study. J Neuroimmunol. (2025) 404:578617. doi: 10.1016/j.jneuroim.2025.578617 40245781

[86] Dong ZY He MJ Yu YK Wang F Zhao PY Ran DL . Integrative genetics and multiomics analysis reveal mechanisms and therapeutic targets in vitiligo highlighting JAK STAT pathway regulation of CTSS. Sci Rep. (2025) 15:2245. doi: 10.1038/s41598-025-86134-4 39824912 PMC11742684

[87] Jiang Y Huang Y Ma G Liu T Li Q Wu H . Granulomatous rosacea in Chinese patients: Clinical-histopathological analysis and pathogenesis exploration. J Dermatol. (2023) 50:856–68. doi: 10.1111/1346-8138.16767 37020415

[88] Viode C Lejeune O Turlier V Rouquier A Casas C Mengeaud V . Cathepsin S, a new pruritus biomarker in clinical dandruff/seborrhoeic dermatitis evaluation. Exp Dermatol. (2014) 23:274–5. doi: 10.1111/exd.12357 24690038

[89] Genies C Maitre M Baradat S Froliger M Turlier V Mercadie A . Metabolomics in scalp seborrheic dermatitis evidenced key changes in inflammatory markers following a 10-week scalp care scheme with an anti-dandruff shampoo. Dermatol Ther (Heidelb). (2026) 16:1647–64. doi: 10.1007/s13555-026-01658-5 41680533 PMC13013722

[90] Lee J Jang S Choi M Kang M Lim SG Kim SY . Overexpression of cathepsin S exacerbates lupus pathogenesis through upregulation TLR7 and IFN-alpha in transgenic mice. Sci Rep. (2021) 11:16348. doi: 10.1038/s41598-021-94855-5 34381063 PMC8357804

[91] Vlachogiannis NI Tual-Chalot S Zormpas E Bonini F Ntouros PA Pappa M . Adenosine-to-inosine RNA editing contributes to type I interferon responses in systemic sclerosis. J Autoimmun. (2021) 125:102755. doi: 10.1016/j.jaut.2021.102755 34857436 PMC8713031

[92] Tato M Kumar SV Liu Y Mulay SR Moll S Popper B . Cathepsin S inhibition combines control of systemic and peripheral pathomechanisms of autoimmune tissue injury. Sci Rep. (2017) 7:2775. doi: 10.1038/s41598-017-01894-y 28584258 PMC5459853

[93] Rupanagudi KV Kulkarni OP Lichtnekert J Darisipudi MN Mulay SR Schott B . Cathepsin S inhibition suppresses systemic lupus erythematosus and lupus nephritis because cathepsin S is essential for MHC class II-mediated CD4 T cell and B cell priming. Ann Rheum Dis. (2015) 74:452–63. doi: 10.1136/annrheumdis-2013-203717 24300027

[94] Yen TH Ho WJ Yeh YH Lai YJ . Cathepsin S inhibition suppresses experimental systemic lupus erythematosus-associated pulmonary arterial remodeling. Int J Mol Sci. (2022) 23:12316. doi: 10.3390/ijms232012316 36293172 PMC9603876

[95] Thanei S Theron M Silva AP Reis B Branco L Schirmbeck L . Cathepsin S inhibition suppresses autoimmune-triggered inflammatory responses in macrophages. Biochem Pharmacol. (2017) 146:151–64. doi: 10.1016/j.bcp.2017.10.001 28987592

[96] Toyama S Yamashita T Saigusa R Miura S Nakamura K Hirabayashi M . Decreased serum cathepsin S levels in patients with systemic sclerosis-associated interstitial lung disease. J Dermatol. (2020) 47:1027–32. doi: 10.1111/1346-8138.15458 32515028

[97] Li Z Zhang Y Yang K Zheng D Chen Y Zhang Y . Monocyte-derived LGMN(+) macrophages divert lung injury outcomes toward fibrosis through matrix remodeling. Res (Wash D C). (2026) 9:1341. doi: 10.34133/research.1341 42375546 PMC13311260

[98] Panwar P Hedtke T Heinz A Andrault PM Hoehenwarter W Granville DJ . Expression of elastolytic cathepsins in human skin and their involvement in age-dependent elastin degradation. Biochim Biophys Acta Gen Subj. (2020) 1864:129544. doi: 10.1016/j.bbagen.2020.129544 32007579

[99] Balsrud P Ulven SM Christensen JJ Ottestad I Holven KB . Inflammatory markers and frailty in home-dwelling elderly, a cross-sectional study. BMC Geriatr. (2024) 24:175. doi: 10.1186/s12877-024-04690-2 38373890 PMC10877806

[100] Shi GP Sukhova GK Kuzuya M Ye Q Du J Zhang Y . Deficiency of the cysteine protease cathepsin S impairs microvessel growth. Circ Res. (2003) 92:493–500. doi: 10.1161/01.RES.0000060485.20318.96 12600886

[101] Wang B Sun J Kitamoto S Yang M Grubb A Chapman HA . Cathepsin S controls angiogenesis and tumor growth via matrix-derived angiogenic factors. J Biol Chem. (2006) 281:6020–9. doi: 10.1074/jbc.M509134200 16365041

[102] Oshima E Hayashi Y Xie Z Sato H Hitomi S Shibuta I . M2 macrophage-derived cathepsin S promotes peripheral nerve regeneration via fibroblast-Schwann cell-signaling relay. J Neuroinflamm. (2023) 20:258. doi: 10.1186/s12974-023-02943-2 37946211 PMC10636844

[103] Memmert S Nokhbehsaim M Damanaki A Nogueira AVB Papadopoulou AK Piperi C . Role of cathepsin S In periodontal wound healing-an *in vitro* study on human PDL cells. BMC Oral Health. (2018) 18:60. doi: 10.1186/s12903-018-0518-2 29622023 PMC5887187

[104] Taggart CC Lowe GJ Greene CM Mulgrew AT O'Neill SJ Levine RL . Cathepsin B, L, and S cleave and inactivate secretory leucoprotease inhibitor. J Biol Chem. (2001) 276:33345–52. doi: 10.1074/jbc.M103220200 11435427

[105] Lecaille F Naudin C Sage J Joulin-Giet A Courty A Andrault PM . Specific cleavage of the lung surfactant protein A by human cathepsin S may impair its antibacterial properties. Int J Biochem Cell Biol. (2013) 45:1701–9. doi: 10.1016/j.biocel.2013.05.018 23707200

[106] Rogan MP Taggart CC Greene CM Murphy PG O'Neill SJ McElvaney NG . Loss of microbicidal activity and increased formation of biofilm due to decreased lactoferrin activity in patients with cystic fibrosis. J Infect Dis. (2004) 190:1245–53. doi: 10.1086/423821 15346334

[107] Reinholz M Ruzicka T Schauber J . Cathelicidin LL-37: an antimicrobial peptide with a role in inflammatory skin disease. Ann Dermatol. (2012) 24:126–35. doi: 10.5021/ad.2012.24.2.126 22577261 PMC3346901

[108] Chieosilapatham P Ogawa H Niyonsaba F . Current insights into the role of human beta-defensins in atopic dermatitis. Clin Exp Immunol. (2017) 190:155–66. doi: 10.1111/cei.13013 28708318 PMC5629447

[109] Kwiecinska P Grygier B Morytko A Sanecka-Duin A Majchrzak-Gorecka M Kwitniewski M . Secretory leukocyte protease inhibitor regulates nerve reflex-mediated skin barrier function in psoriasis. J Eur Acad Dermatol Venereol. (2022) 36:1266–74. doi: 10.1111/jdv.18065 35279880 PMC9546283

[110] Ashcroft GS Lei K Jin W Longenecker G Kulkarni AB Greenwell-Wild T . Secretory leukocyte protease inhibitor mediates non-redundant functions necessary for normal wound healing. Nat Med. (2000) 6:1147–53. doi: 10.1038/80489 11017147

[111] Kehlet SN Bager CL Willumsen N Dasgupta B Brodmerkel C Curran M . Cathepsin-S degraded decorin are elevated in fibrotic lung disorders - development and biological validation of a new serum biomarker. BMC Pulm Med. (2017) 17:110. doi: 10.1186/s12890-017-0455-x 28793886 PMC5550991

[112] Zhang P Hou S Chen J Zhang J Lin F Ju R . Smad4 deficiency in smooth muscle cells initiates the formation of aortic aneurysm. Circ Res. (2016) 118:388–99. doi: 10.1161/CIRCRESAHA.115.308040 26699655

[113] Chen H Wang J Xiang MX Lin Y He A Jin CN . Cathepsin S-mediated fibroblast trans-differentiation contributes to left ventricular remodelling after myocardial infarction. Cardiovasc Res. (2013) 100:84–94. doi: 10.1093/cvr/cvt158 23771947 PMC3778959

[114] Qin Y Cao X Guo J Zhang Y Pan L Zhang H . Deficiency of cathepsin S attenuates angiotensin II-induced abdominal aortic aneurysm formation in apolipoprotein E-deficient mice. Cardiovasc Res. (2012) 96:401–10. doi: 10.1093/cvr/cvs263 22871592 PMC3500043

[115] Yao X Cheng F Yu W Rao T Li W Zhao S . Cathepsin S regulates renal fibrosis in mouse models of mild and severe hydronephrosis. Mol Med Rep. (2019) 20:141–50. doi: 10.3892/mmr.2019.10230 31115520 PMC6580002

[116] Zhou L Zhou Q Guo Q Lai P Rui C Li W . Dual role of Cathepsin S in cutaneous melanoma: insights from mendelian randomization and bioinformatics analysis. BMC Cancer. (2025) 25:104. doi: 10.1186/s12885-025-13481-w 39833734 PMC11744831

[117] Kim S Jin H Seo HR Lee HJ Lee YS . Regulating BRCA1 protein stability by cathepsin S-mediated ubiquitin degradation. Cell Death Differ. (2019) 26:812–25. doi: 10.1038/s41418-018-0153-0 30006610 PMC6461859

[118] Liang S Dang B Chen S Mi H . Prognostic value and immunological role of cathepsin S gene in pan-cancer. Oncol Lett. (2024) 27:41. doi: 10.3892/ol.2023.14175 38108072 PMC10722551

[119] Hsin MC Hsieh YH Wang PH Ko JL Hsin IL Yang SF . Hispolon suppresses metastasis via autophagic degradation of cathepsin S in cervical cancer cells. Cell Death Dis. (2017) 8:e3089. doi: 10.1038/cddis.2017.459 28981104 PMC5680581

[120] Dheilly E Battistello E Katanayeva N Sungalee S Michaux J Duns G . Cathepsin S regulates antigen processing and T cell activity in non-hodgkin lymphoma. Cancer Cell. (2020) 37:674–89:e12. doi: 10.1016/j.ccell.2020.03.016 32330455

[121] Kremenovic M Chan AA Feng B Bariswyl L Robatel S Gruber T . BCG hydrogel promotes CTSS-mediated antigen processing and presentation, thereby suppressing metastasis and prolonging survival in melanoma. J Immunother Cancer. (2022) 10:e004133. doi: 10.1136/jitc-2021-004133 35732347 PMC9226922

[122] Thomas NE Busam KJ From L Kricker A Armstrong BK Anton-Culver H . Tumor-infiltrating lymphocyte grade in primary melanomas is independently associated with melanoma-specific survival in the population-based genes, environment and melanoma study. J Clin Oncol. (2013) 31:4252–9. doi: 10.1200/JCO.2013.51.3002 24127443 PMC3821014

[123] Barclay J Clark AK Ganju P Gentry C Patel S Wotherspoon G . Role of the cysteine protease cathepsin S in neuropathic hyperalgesia. Pain. (2007) 130:225–34. doi: 10.1016/j.pain.2006.11.017 17250968

[124] Clark AK Yip PK Grist J Gentry C Staniland AA Marchand F . Inhibition of spinal microglial cathepsin S for the reversal of neuropathic pain. Proc Natl Acad Sci USA. (2007) 104:10655–60. doi: 10.1073/pnas.0610811104 17551020 PMC1965568

[125] Clark AK Staniland AA Malcangio M . Fractalkine/CX3CR1 signalling in chronic pain and inflammation. Curr Pharm Bio/Technol. (2011) 12:1707–14. doi: 10.2174/138920111798357465 21466443

[126] Clark AK Grist J Al-Kashi A Perretti M Malcangio M . Spinal cathepsin S and fractalkine contribute to chronic pain in the collagen-induced arthritis model. Arthritis Rheum. (2012) 64:2038–47. doi: 10.1002/art.34351 22213084

[127] Clark AK Yip PK Malcangio M . The liberation of fractalkine in the dorsal horn requires microglial cathepsin S. J Neurosci. (2009) 29:6945–54. doi: 10.1523/JNEUROSCI.0828-09.2009 19474321 PMC2698289

[128] Clark AK Malcangio M . Microglial signalling mechanisms: Cathepsin S and Fractalkine. Exp Neurol. (2012) 234:283–92. doi: 10.1016/j.expneurol.2011.09.012 21946268

[129] Zhuang ZY Kawasaki Y Tan PH Wen YR Huang J Ji RR . Role of the CX3CR1/p38 MAPK pathway in spinal microglia for the development of neuropathic pain following nerve injury-induced cleavage of fractalkine. Brain Behav Immun. (2007) 21:642–51. doi: 10.1016/j.bbi.2006.11.003 17174525 PMC2084372

[130] Bonfante R Napimoga MH Macedo CG Abdalla HB Pieroni V Clemente-Napimoga JT . The P2X7 receptor, cathepsin S and fractalkine in the trigeminal subnucleus caudalis signal persistent hypernociception in temporomandibular rat joints. Neuroscience. (2018) 391:120–30. doi: 10.1016/j.neuroscience.2018.09.005 30248434

[131] Szukiewicz D . CX3CL1 (Fractalkine)-CX3CR1 axis in inflammation-induced angiogenesis and tumorigenesis. Int J Mol Sci. (2024) 25:4679. doi: 10.3390/ijms25094679 38731899 PMC11083509

[132] Liu PP Liu XH Ren MJ Liu XT Shi XQ Li ML . Neuronal cathepsin S increases neuroinflammation and causes cognitive decline via CX3CL1-CX3CR1 axis and JAK2-STAT3 pathway in aging and Alzheimer's disease. Aging Cell. (2025) 24:e14393. doi: 10.1111/acel.14393 39453382 PMC11822647

[133] Finneran D Li Q Subbarayan MS Joly-Amado A Kamath S Dengler DG . Concentration and proteolysis of CX3CL1 may regulate the microglial response to CX3CL1. Glia. (2023) 71:245–58. doi: 10.1002/glia.24269 36106533 PMC9772123

[134] Jimenez-Vargas NN Pattison LA Zhao P Lieu T Latorre R Jensen DD . Protease-activated receptor-2 in endosomes signals persistent pain of irritable bowel syndrome. Proc Natl Acad Sci USA. (2018) 115:E7438–E47. doi: 10.1073/pnas.1721891115 30012612 PMC6077730

[135] Tu NH Inoue K Chen E Anderson BM Sawicki CM Scheff NN . Cathepsin S evokes PAR(2)-dependent pain in oral squamous cell carcinoma patients and preclinical mouse models. Cancers (Basel). (2021) 13:4697. doi: 10.3390/cancers13184697 34572924 PMC8466361

[136] Irie O Kosaka T Ehara T Yokokawa F Kanazawa T Hirao H . Discovery of orally bioavailable cathepsin S inhibitors for the reversal of neuropathic pain. J Med Chem. (2008) 51:5502–5. doi: 10.1021/jm800839j 18754655

[137] Chang Z Zhao G Zhao Y Lu H Xiong W Liang W . BAF60a deficiency in vascular smooth muscle cells prevents abdominal aortic aneurysm by reducing inflammation and extracellular matrix degradation. Arterioscler Thromb Vasc Biol. (2020) 40:2494–507. doi: 10.1161/ATVBAHA.120.314955 32787523 PMC7530061

[138] Gadola SD Farber P Posch MG Nagel S Canducci F . An open-label phase 2a study investigating the efficacy and safety of a cathepsin S inhibitor in patients with moderate-to-severe psoriasis. J Am Acad Dermatol. (2022) 87:1089–91. doi: 10.1016/j.jaad.2021.09.074 34906661

[139] Feth MP Heyse W Baumgartner B Nagel N Tappertzhofen C Olpp T . From laboratory to pilot plant: the solid-state process development of a highly potent cathepsin S/K inhibitor. Eur J Pharm Biopharm. (2013) 83:436–48. doi: 10.1016/j.ejpb.2012.11.007 23201054

[140] Bentley D Fisher BA Barone F Kolb FA Attley G . A randomized, double-blind, placebo-controlled, parallel group study on the effects of a cathepsin S inhibitor in primary Sjogren's syndrome. Rheumatol (Oxford). (2023) 62:3644–53. doi: 10.1093/rheumatology/kead092 36864622 PMC10629789

[141] Vizovisek M Vidmar R Van Quickelberghe E Impens F Andjelkovic U Sobotic B . Fast profiling of protease specificity reveals similar substrate specificities for cathepsins K, L and S. Proteomics. (2015) 15:2479–90. doi: 10.1002/pmic.201400460 25626674

[142] Burden RE Snoddy P Buick RJ Johnston JA Walker B Scott CJ . Recombinant cathepsin S propeptide attenuates cell invasion by inhibition of cathepsin L-like proteases in tumor microenvironment. Mol Cancer Ther. (2008) 7:538–47. doi: 10.1158/1535-7163.MCT-07-0528 18347141

[143] Guay J Falgueyret JP Ducret A Percival MD Mancini JA . Potency and selectivity of inhibition of cathepsin K, L and S by their respective propeptides. Eur J Biochem. (2000) 267:6311–8. doi: 10.1046/j.1432-1327.2000.01730.x 11012686

[144] Barry ZT Platt MO . Cathepsin S cannibalism of cathepsin K as a mechanism to reduce type I collagen degradation. J Biol Chem. (2012) 287:27723–30. doi: 10.1074/jbc.M111.332684 22730330 PMC3431690

[145] Pecar Fonovic U Kos J Mitrovic A . Compensational role between cathepsins. Biochimie. (2024) 226:62–76. doi: 10.1016/j.biochi.2024.04.010 38663456

[146] Fuchs N Meta M Schuppan D Nuhn L Schirmeister T . Novel opportunities for cathepsin S inhibitors in cancer immunotherapy by nanocarrier-mediated delivery. Cells. (2020) 9:2021. doi: 10.3390/cells9092021 32887380 PMC7565055

[147] DeHority R Gil Pineda LI Cochran K Chen B Bratek D Helm RF . The pH-dependent specificity of cathepsin S and its implications for inflammatory communications and disease. Biochemistry. (2025) 64:3841–53. doi: 10.1021/acs.biochem.5c00287 40682537 PMC12444987

[148] Smyth P Ferguson L Burrows JF Burden RE Tracey SR Herron UM . Evaluation of variable new antigen receptors (vNARs) as a novel cathepsin S (CTSS) targeting strategy. Front Pharmacol. (2023) 14:1296567. doi: 10.3389/fphar.2023.1296567 38116078 PMC10728302

[149] Nguyen DD Cang Z Wu K Wang M Cao Y Wei GW . Mathematical deep learning for pose and binding affinity prediction and ranking in D3R Grand Challenges. J Comput-Aided Mol Des. (2019) 33:71–82. doi: 10.1007/s10822-018-0146-6 30116918 PMC7163798

[150] Wu H Huang W Cai X He H Wang Y Yao S . Hydroxybutyl chitosan hydrogel promotes ablative fractional CO(2) laser wound healing: A prospective, randomized, split-face trial and animal model evidence. Lasers Surg Med. (2026) 58:211–22. doi: 10.1002/lsm.70103 41607028

[151] Ajani TA Magwebu ZE Chauke CG Obikeze K . Advances in cathepsin S inhibition: Challenges and breakthroughs in drug development. Pathophysiology. (2024) 31:471–87. doi: 10.3390/pathophysiology31030035 39311309 PMC11417842

[152] Bentley D Mannino M Manchester M Teixeira PC Reis B Boyce M . A randomized, double-blind, placebo-controlled, multiple dose, parallel study to investigate the effects of a cathepsin S inhibitor in celiac disease. Clin Transl Sci. (2025) 18:e13901. doi: 10.1111/cts.13901 39739628 PMC11686337

[153] Hargreaves P Daoudlarian D Theron M Kolb FA Manchester Young M Reis B . Differential effects of specific cathepsin S inhibition in biocompartments from patients with primary Sjogren syndrome. Arthritis Res Ther. (2019) 21:175. doi: 10.1186/s13075-019-1955-2 31319889 PMC6637481

[154] Payne CD Deeg MA Chan M Tan LH LaBell ES Shen T . Pharmacokinetics and pharmacodynamics of the cathepsin S inhibitor, LY3000328, in healthy subjects. Br J Clin Pharmacol. (2014) 78:1334–42. doi: 10.1111/bcp.12470 25039273 PMC4256622

[155] Cianni L Feldmann CW Gilberg E Gutschow M Juliano L Leitao A . Can cysteine protease cross-class inhibitors achieve selectivity? J Med Chem. (2019) 62:10497–525. doi: 10.1021/acs.jmedchem.9b00683 31361135

[156] Orellano LAA Zeng C Zhu J Bogyo M Rock KL Lai JJ . A broad cathepsin inhibitor blocks crystal-stimulated inflammasome-dependent and -independent inflammation and gout arthritis. Commun Med (Lond). (2026) 6:345. doi: 10.1038/s43856-026-01590-x 41998291 PMC13273054

[157] Baugh M Black D Westwood P Kinghorn E McGregor K Bruin J . Therapeutic dosing of an orally active, selective cathepsin S inhibitor suppresses disease in models of autoimmunity. J Autoimmun. (2011) 36:201–9. doi: 10.1016/j.jaut.2011.01.003 21439785

[158] Hewitt E Pitcher T Rizoska B Tunblad K Henderson I Sahlberg BL . Selective cathepsin S inhibition with MIV-247 attenuates mechanical allodynia and enhances the antiallodynic effects of gabapentin and pregabalin in a mouse model of neuropathic pain. J Pharmacol Exp Ther. (2016) 358:387–96. doi: 10.1124/jpet.116.232926 27335437

